# *PARK2* Depletion Connects Energy and Oxidative Stress to PI3K/Akt Activation via PTEN S-Nitrosylation

**DOI:** 10.1016/j.molcel.2017.02.019

**Published:** 2017-03-16

**Authors:** Amit Gupta, Sara Anjomani-Virmouni, Nikos Koundouros, Maria Dimitriadi, Rayman Choo-Wing, Adamo Valle, Yuxiang Zheng, Yu-Hsin Chiu, Sameer Agnihotri, Gelareh Zadeh, John M. Asara, Dimitrios Anastasiou, Mark J. Arends, Lewis C. Cantley, George Poulogiannis

**Affiliations:** 1 Signalling and Cancer Metabolism Team, Division of Cancer Biology, The Institute of Cancer Research, 237 Fulham Road, London SW3 6JB, UK; 2 Division of Computational and Systems Medicine, Department of Surgery and Cancer, Imperial College London, London SW7 2AZ, UK; 3 Department of Biological and Environmental Sciences, University of Hertfordshire, Hatfield AL10 9AB, UK; 4 Novartis Institutes for BioMedical Research, Inc., 181 Massachusetts Avenue, Cambridge, MA 02139, USA; 5 Energy Metabolism and Nutrition, University of Balearic Islands, Research Institute of Health Sciences (IUNICS) and Medical Research Institute of Palma (IdISPa), 07122 Palma de Mallorca, Spain; 6 Biomedical Research Networking Center for Physiopathology of Obesity and Nutrition (CIBERobn), Instituto de Salud Carlos III, 28029 Madrid, Spain; 7 Meyer Cancer Center, Department of Medicine, Weill Cornell Medicine, New York, NY 10065, USA; 8 Novartis Institutes for BioMedical Research, Inc., 22 Windsor Street, Cambridge, MA 02139, USA; 9 MacFeeters-Hamilton Neurooncology Program, Princess Margaret Cancer Centre, Toronto, ON M5G 2M9, Canada; 10 Division of Signal Transduction, Beth Israel Deaconess Medical Center, Boston, MA 02115, USA; 11 Department of Medicine, Harvard Medical School, Boston, MA 02175, USA; 12 Cancer Metabolism Laboratory, The Francis Crick Institute, London NW7 1AA, UK; 13 University of Edinburgh, Division of Pathology, Edinburgh Cancer Research Centre, Institute of Genetics & Molecular Medicine, Western General Hospital, Edinburgh EH4 2XR, UK

**Keywords:** *PARK2,* PI3K/Akt activation, AMPK, nitric oxide, *PTEN*, S-nitrosylation

## Abstract

*PARK2* is a gene implicated in disease states with opposing responses in cell fate determination, yet its contribution in pro-survival signaling is largely unknown. Here we show that *PARK2* is altered in over a third of all human cancers, and its depletion results in enhanced phosphatidylinositol 3-kinase/Akt (PI3K/Akt) activation and increased vulnerability to PI3K/Akt/mTOR inhibitors. *PARK2* depletion contributes to AMPK-mediated activation of endothelial nitric oxide synthase (eNOS), enhanced levels of reactive oxygen species, and a concomitant increase in oxidized nitric oxide levels, thereby promoting the inhibition of PTEN by S-nitrosylation and ubiquitination. Notably, AMPK activation alone is sufficient to induce PTEN S-nitrosylation in the absence of *PARK2* depletion. *Park2* loss and *Pten* loss also display striking cooperativity to promote tumorigenesis in vivo. Together, our findings reveal an important missing mechanism that might account for PTEN suppression in *PARK2*-deficient tumors, and they highlight the importance of PTEN S-nitrosylation in supporting cell survival and proliferation under conditions of energy deprivation.

## Highlights

• *PARK2* negatively regulates the PI3K/Akt pathway
• *PARK2* depletion promotes PTEN inactivation by S-nitrosylation and ubiquitination
• AMPK activation triggers PTEN S-nitrosylation in the absence of *PARK2* depletion
• *PARK2* loss and *PTEN* loss display striking cooperativity to promote tumorigenesis in vivo

## Graphical Abstract

**Figure:**
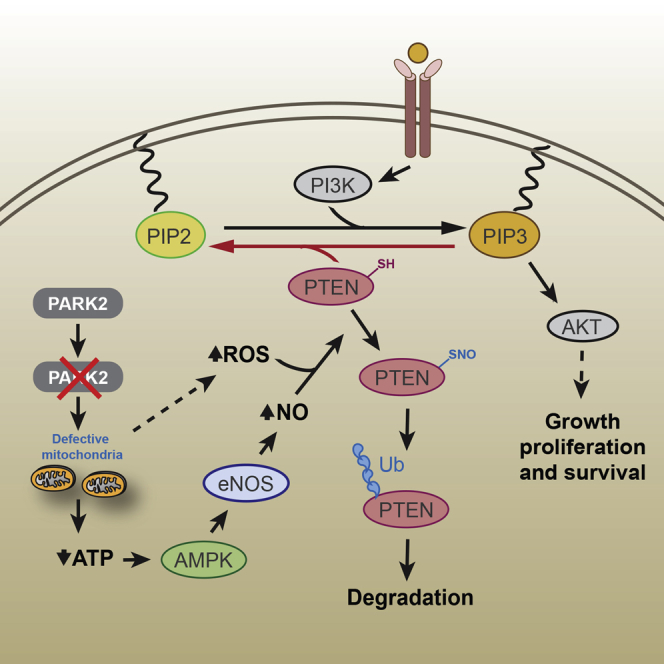

## Teaser

Gupta et al. reveal an important missing piece in the dynamic signaling and metabolic network governing PI3K/Akt activation. *PARK2* inactivation connects energy and oxidative stress to Akt activation via redox-mediated inactivation of PTEN by S-nitrosylation to support cell survival under conditions of energy deprivation.

## Introduction

Cell homeostasis is achieved through an orchestrated balance of cell signaling interactions that dictate the likelihood of a cell to escape from normal growth restraints or be eliminated from the replicative pool, resulting in cell death. Cancer is a group of diseases that are due to escape from cell death control, while Parkinson’s disease (PD) portrays a disease that results from accelerated cell death. It would seem unlikely that these diseases are related, yet there is increasing evidence to suggest that a subset of PD susceptibility genes are also associated with cancer (Ong et al., 2014).

*PARK2* was originally identified as a gene associated with the pathogenesis of familial PD in early-onset autosomal recessive juvenile parkinsonism (Kitada et al., 1998). It has been reported to be mutated in as high as 77% of PD patients with an age of onset of <20 years, but only in 3% of patients with an age of onset of >30 years (Lücking et al., 2000). Subsequently, it has been linked to a wide range of disorders, including leprosy (Mira et al., 2004), autism (Glessner et al., 2009), type 2 diabetes mellitus (Wongseree et al., 2009), Alzheimer’s disease (Burns et al., 2009), cerebellar ataxia (Periquet et al., 2003), resistance to intracellular pathogen infections (Manzanillo et al., 2013), and cancer, where it is somatically deleted in a wide spectrum of tumor types (Bernardini et al., 2016). *PARK2* is a bona fide haploinsufficient tumor suppressor, as depletion of a single *PARK2* allele significantly increases adenoma development and polyp multiplicity in Apc^Min/+^ mice (Poulogiannis et al., 2010). *PARK2* loss also renders mice more susceptible to hepatocellular (Fujiwara et al., 2008) and γ-irradiation-induced carcinomas (Zhang et al., 2011), while ectopic *PARK2* expression mitigates cell proliferation in colorectal, glioma, breast, hepatocellular, and non-small-cell lung cancer cells (Lin et al., 2015, Picchio et al., 2004, Poulogiannis et al., 2010, Tay et al., 2010, Veeriah et al., 2010, Wang et al., 2004, Yeo et al., 2012).

The *PARK2* gene encodes the E3 ubiquitin ligase Parkin, which mediates the ubiquitination of a number of substrate proteins, leading to their proteasomal degradation (Dawson and Dawson, 2010). Its activities go beyond the degradative ubiquitination, and it is implicated in the regulation of multiple cellular processes, including stress response, mitochondrial biogenesis, and stability of G1/S cyclins (Corti and Brice, 2013, Gong et al., 2014). Although the underlying mechanisms by which pathogenic *PARK2* mutations contribute to PD are not entirely understood, mitochondrial dysfunction is considered to play a central role in stress-induced neuronal cell death associated with the pathogenesis of this disorder. Increased oxidative and nitrosative stress is a common phenomenon in both PD and cancer; hence, it is imperative to identify the molecular pathways underlying the functional contribution of *PARK2* depletion in these processes.

Compelling evidence shows that cancer cells utilize multiple pathways, including the phosphatidylinositol 3-kinase/Akt (PI3K/Akt) signaling pathway, to enhance their survival and prevent apoptosis under metabolic stress conditions (Trachootham et al., 2008). Importantly, *PARK2* has previously been associated with the activation of the Akt pathway (Fallon et al., 2006, Lin et al., 2015, Yeo et al., 2012); however, the mechanistic evidence behind its functional contribution is unclear. One study showed that Parkin interacts with and ubiquitinates Eps15 to delay the internalization and degradation of its adaptor protein epidermal growth factor receptor (EGFR), thereby promoting PI3K/Akt signaling (Fallon et al., 2006), while a more recent study suggests that Parkin directly interacts with and promotes the ubiquitination of EGFR, leading to diminished activation of EGF-induced PI3K/Akt signaling (Lin et al., 2015). These data highlight the need for further investigation of the molecular events underlying the role of *PARK2* depletion in PI3K/Akt-mediated cellular survival.

In this study, we identified PTEN as an important mediator behind the functional contribution of *PARK2* depletion in the activation of the PI3K/Akt pathway, and we further characterized its pivotal role in the tumor suppressor function of *PARK2* in vitro and in vivo. Additionally, our results reveal an important missing piece in the dynamic signaling and metabolic network connecting AMPK with Akt activation in the absence of mTORC1-S6K-dependent negative feedback loop mechanisms (Efeyan and Sabatini, 2010), demonstrating a compensatory survival mechanism for cancer cells under conditions of energy deprivation.

## Results

### *PARK2* Genomic and Gene Expression Profiling across Human Cancers

**Figure 1:**
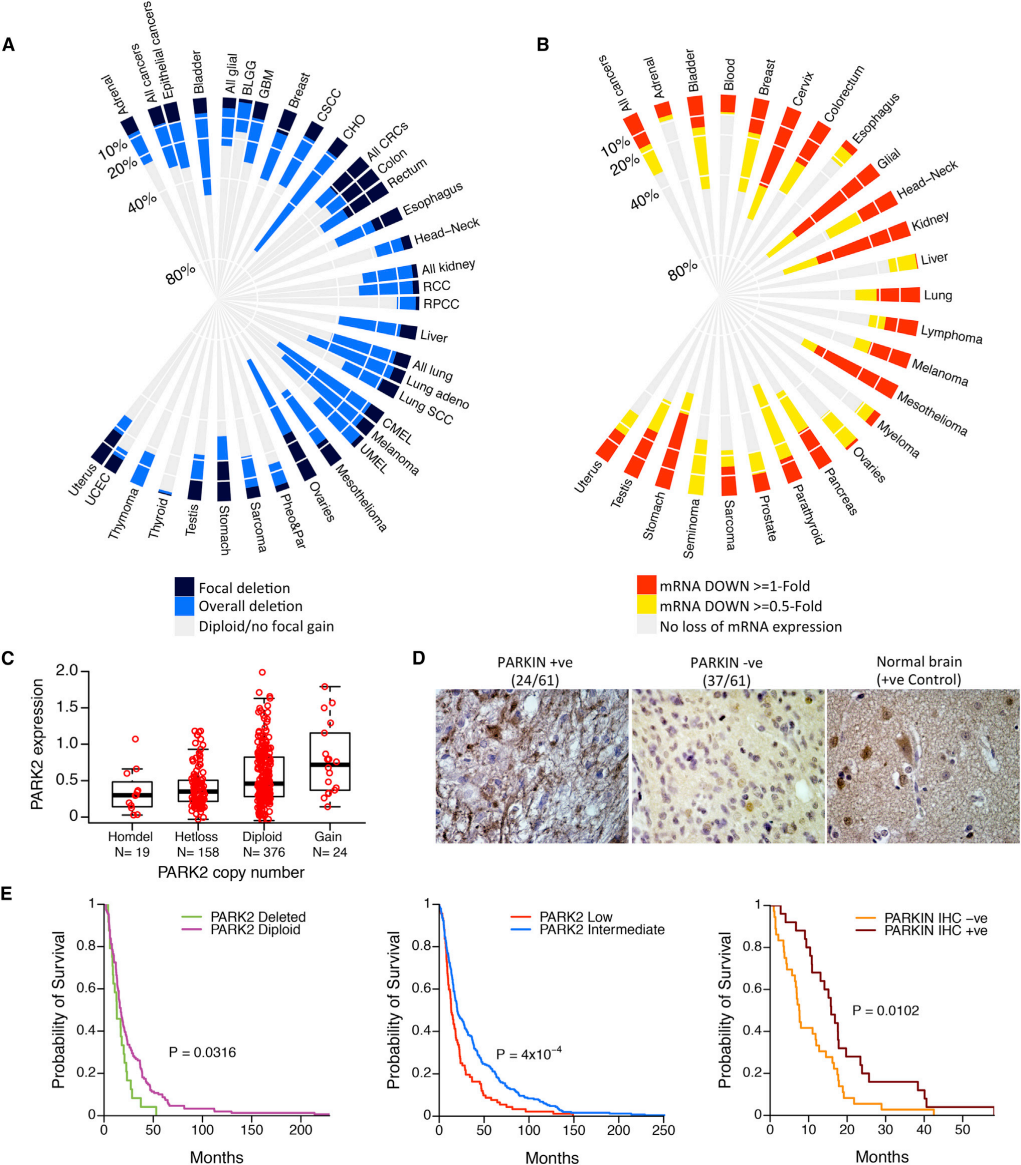
Genetic Landscape of *PARK2* Copy Number and mRNA Expression across Human Cancers (A) Frequencies of *PARK2* deletion from 9,863 primary tumors across 28 different cancer types. Colored bars describe the percentage of tumor samples showing focal deletion (dark blue), overall deletion (light blue), and diploid/no focal gain (gray). (B) Frequencies of relative fold change of *PARK2* mRNA underexpression between cancerous and corresponding normal tissues (≥0.5- and ≥1-log2-fold change in tumor versus normal). The analysis was performed on 13,481 specimens from 25 different cancer types (mRNA downregulated ≥1-log2-fold change, red; mRNA downregulated ≥0.5-log2-fold, yellow; no loss of mRNA expression, gray). The putative DCN and mRNA expression data for *PARK2* were retrieved from the TCGA database. (C) *PARK2* mRNA expression in subgroups of primary GBMs of different ploidy status, ranging from gain (n = 24) to diploid (n = 376), heterozygous deletion (Hetloss, n = 158), and homozygous deletion (Homdel, n = 19) (p = 3.5 × 10^−7^, one-way ANOVA). (D) Representative IHC staining intensities of PARKIN protein expression across 61 GBM specimens. (E) Kaplan-Meier survival plots of glioma cancer patients stratified by tumors bearing *PARK2* deletion versus retention (left: *PARK2* Deleted ≤1.8 copies, n = 24; *PARK2* Diploid two copies, n = 149), low versus intermediate *PARK2* mRNA expression (middle: *PARK2* Low ≥two times lower than log2 median expression, n = 92; *PARK2* Intermediate <two times lower or higher than log2 median expression, n = 251), and positive versus negative IHC PARKIN protein expression (right) (from left to right: p = 0.032, p = 4 × 10^−4^, p = 0.01, log-rank test). See also Figure S1 and Tables S1 and S2.

We examined the degree of *PARK2* deletion in the largest up-to-date collection of The Cancer Genome Atlas (TCGA), assembling data from 9,863 primary tumors from 28 different tumor types (Table S1). Focal deletions (Figure 1A, dark blue) of the *PARK2* gene were most commonly found in colorectal (21%) and ovarian (25%) carcinomas, while a strikingly high number of tumors, including lung adenocarcinomas, melanomas, bladder, ovarian, and pancreatic, had an overall >40% DNA copy number (DCN) loss of the *PARK2* gene (both focal deletion [Figure 1A, dark blue] and as part of whole or part chromosome arm losses [Figure 1A, light blue]). Cholangiosarcomas (CHO) showed a staggering 69% (25/36) rate of overall *PARK2* deletions. In parallel, we also examined the degree of *PARK2* mRNA decrease between cancer and corresponding normal tissues from a total number of 13,481 specimens, studied in 127 published microarray datasets (Table S2), covering 25 tumor types. Notably, there were many cancer types, including gliomas, cervical, and kidney carcinomas, with not the highest frequency of overall *PARK2* deletion (<40%) but with widespread (up to 69%) decrease or loss of its mRNA expression compared to their normal counterparts (Figures 1B and S1A; >1-log2-fold [red] or >0.5-log2-fold [red and yellow]). Vice versa, there were some tumor types, e.g., ovarian cancer, where *PARK2* deletion was found in >60% of the cases, yet only a small fraction were reported to have low *PARK2* mRNA expression. This reflects in part the stringent criteria we used to report tumor types with >0.5-fold changes in mRNA expression compared to their normal counterparts, but also that the majority (>90%) of *PARK2* deletions across all tumor types are heterozygous and, hence, less likely to cause a marked reduction in *PARK2* mRNA expression.

As high as two-thirds of glioma tumors had significantly reduced levels of *PARK2* mRNA expression compared to their corresponding normal tissues (Figure 1B). We plotted the distribution of *PARK2* expression, across different subgroups of gliomas reminiscent of distinct neural cell types, to show that *PARK2* is significantly downregulated irrespective of the histological origin of this tumor type (Figure S1B). To identify relationships between *PARK2* DCN and mRNA expression levels, we plotted the distribution of its expression across subgroups of glioblastomas (GBMs) with different *PARK2* DCN. *PARK2* mRNA expression was progressively lower across the different subgroups of *PARK2* ploidy status (Figure 1C). To assess the prognostic significance of this gene across different types of genetic alteration, we also performed survival analysis between subgroups of patients with GBM, divided based on different molecular markers of the *PARK2* gene. Interestingly, loss of *PARK2* at the DNA, mRNA, and protein levels (Figure 1D) all correlated with significantly poorer survival in patients with GBM (Figure 1E). Of note, *PARK2* expression was also associated with poorer survival in patients with breast and lung adenocarcinomas (Figures S1C and S1D). Collectively, our data demonstrate that *PARK2* is altered in over a third of all human cancers. Its widespread loss or decrease across many molecular biomarker indicators (DNA, mRNA, and protein) significantly correlates with poorer survival, offering great prognostic and predictive value for clinical practice.

### *PARK2* Regulates the Activation of the PI3K/Akt Pathway

**Figure 2:**
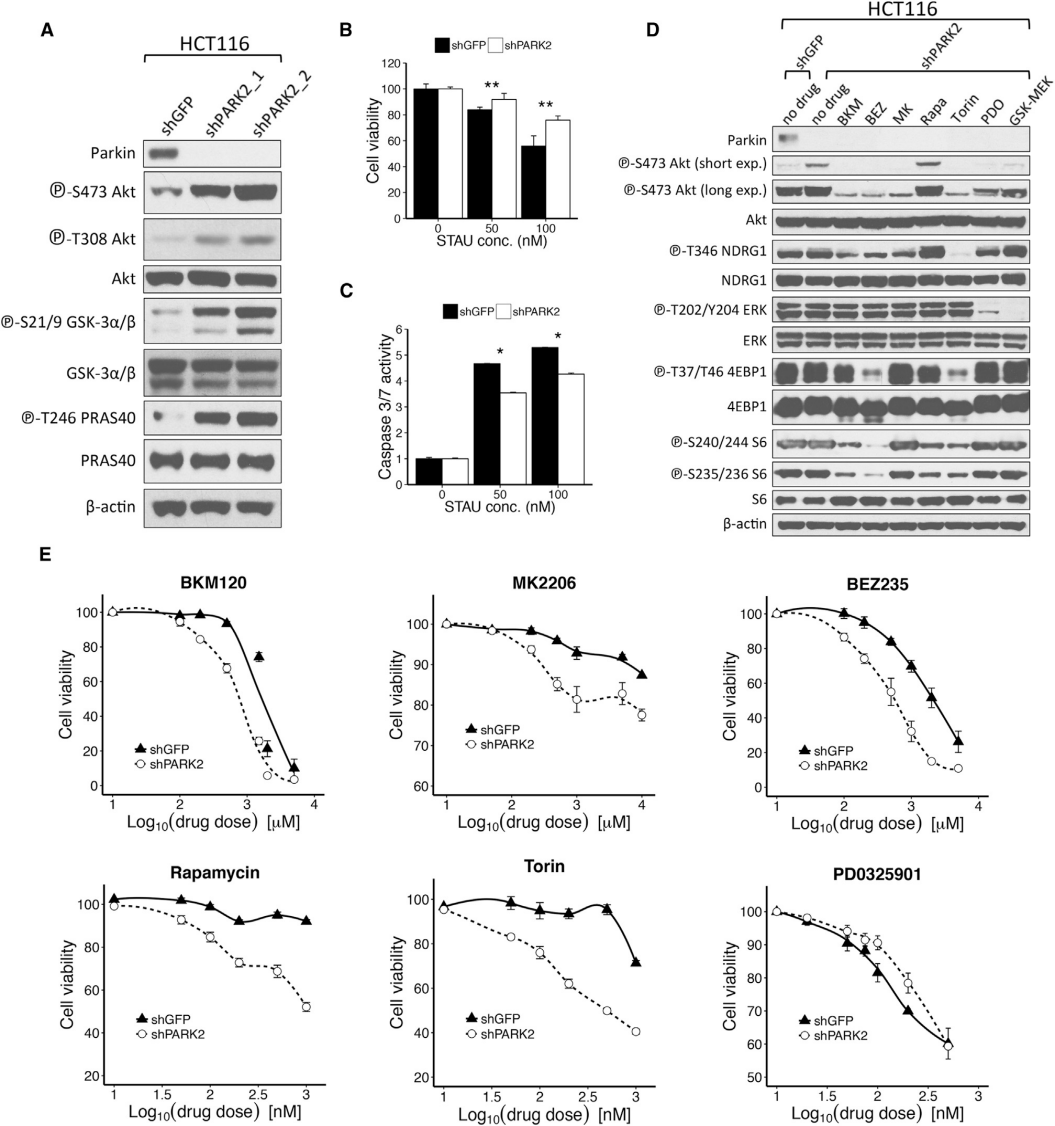
*PARK2* Depletion Contributes to the Activation of the PI3K/Akt Pathway (A) Immunoblotting analysis of HCT116 cells stably transfected with control *GFP* (shGFP) or *PARK2* (shPARK2_1 and shPARK2_2) lentiviral hairpins. (B and C) Shown is the (B) CellTiter 96 AQueous Non-Radioactive Cell Proliferation (MTS) assay and (C) caspase-3/7 activity assay of shGFP or shPARK2 HCT116 cells, following treatment with increasing concentrations of staurosporine (50 and 100 nM) for 1 hr (MTS assay, p = 0.001 for 50-nM and p = 0.0008 for 100-nM treatment; caspase-3/7 assay, p = 0.043 for 50-nM and p = 0.036 for 100-nM treatment). (D) Immunoblotting analysis of shGFP and shPARK2 HCT116 cells following treatment with the indicated compounds: 1 μM BKM (NVP-BKM120) or 500 nM BEZ (NVP-BEZ235) for 24 hr, 500 nM MK (MK 2206) for 4 hr, 100 nM Rapa (Rapamycin) or Torin for 2 hr, 100 nM PD0 (PD0325901) for 1 hr, or 10 nM GSK (GSK1120212) for 6 hr. (E) Drug dose-response curves of shGFP and shPARK2 HCT116 cells treated with the indicated compounds for 24 hr (NVP-BKM120, p = 0.014; NVP-BEZ235, p = 9.28 × 10^−5^; MK 2206, p = 1 × 10^−4^; Rapamycin, p = 1.45 × 10^−11^; Torin, p = 6.73 × 10^−10^; PD0325901, p = 0.02; and GSK1120212, p = 0.03, two-way ANOVA). Data are represented as mean ± SEM (^∗^p < 0.05 and ^∗∗^p < 0.01, two-tailed t test). See also Figure S2.

The PI3K/Akt pathway is the single most frequently altered signaling cascade across all the tumor types, with recurrent *PARK2* deletions and mRNA loss/reduction (Yuan and Cantley, 2008). Activation of the PI3K/AKT/mTOR pathway has also been associated with significantly poorer survival across many solid tumors (Ocana et al., 2014). To investigate whether activation of this pathway is inherently associated with loss of the *PARK2* gene, we stably expressed two independent hairpins targeted against *PARK2* in HCT116 cells. Cells with *PARK2* knockdown exhibited a pronounced increase in Akt phosphorylation compared to control GFP knockdown cells (Figure 2A). Staurosporine is a protein kinase inhibitor that can induce apoptosis across many different cell types, and Akt hyperactivation has been shown to attenuate sensitivity to staurosporine-induced cell death (Mookherjee et al., 2007). We showed that *PARK2* depletion augmented resistance to staurosporine-induced cell death (Figure 2B), and this was consistent with significantly lower apoptotic response, as indicated by caspase-3/7 activity (Figure 2C).

Identification of candidate biomarkers that predict responsiveness to specific signaling cascade inhibitors is increasingly important in the era of personalized medicine. Accordingly, we assessed the role of *PARK2* knockdown to the inhibition of various signaling nodes across the PI3K/Akt/mTOR and MAPK pathways. *PARK2* depletion rendered cells more sensitive to inhibitors of PI3K (BKM120 and BEZ235), Akt (MK2206), and mTOR (BEZ235, Rapamycin, and Torin), but it decreased the effectiveness of MEK inhibitors PD0325901 and GSK1120212 (Figures 2D, 2E, S2A, and S2B). Taken together, these data suggest that *PARK2* loss contributes in the activation of Akt signaling and the dependence of cells on this pathway, suggesting that *PARK2* deficiency could be used as a biomarker of efficacy and favorable clinical response to inhibitors of the PI3K/Akt/mTOR pathway.

### *PARK2* Suppresses Akt Activation and Tumorigenicity in *PTEN* Wild-Type but Not *PTEN* Mutant Cells

**Figure 3:**
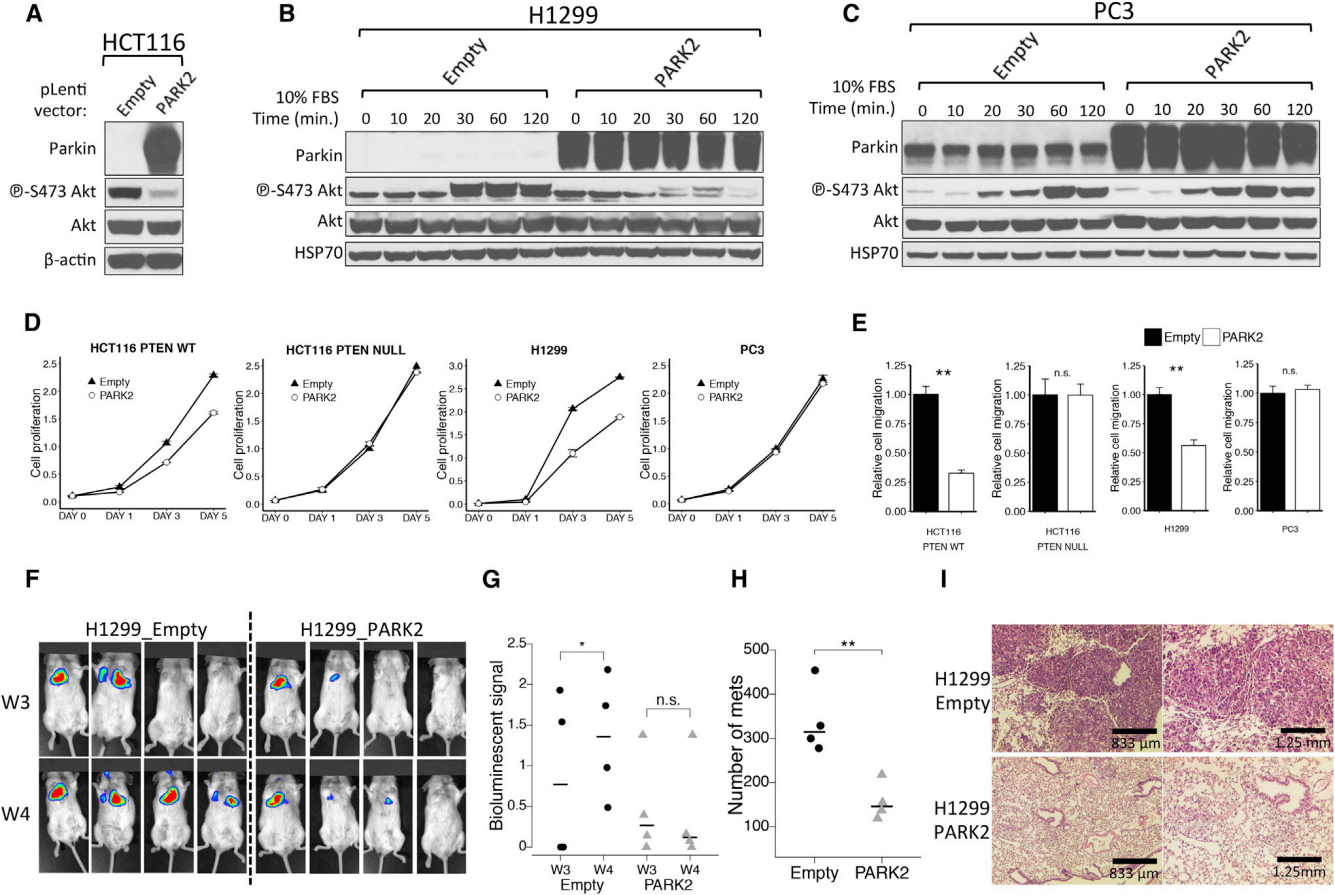
*PARK2* Overexpression Suppresses Akt Activation and Mitigates Cell Proliferation and Migration in *PTEN* Wild-Type, but Not *PTEN* Mutant, Cells (A–C) Immunoblotting analysis of (A) HCT116 cells transfected with vector only or vector encoding WT *PARK2*, (B) *PTEN* WT H1299 cells, and (C) *PTEN* mutant PC3 cells expressing vector only or vector encoding WT *PARK2* following 10% fetal bovine serum (FBS) stimulation. Cells were serum-starved for 24 hr prior to FBS stimulation. (D and E) Shown are the (D) cell proliferation (HCT116 *PTEN*^+/+^, p = 0.002; HCT116 *PTEN*^−/−^, p = 0.99; H1299, p = 3.4 × 10^−5^; and PC3, p = 0.66, two-way ANOVA) and (E) cell migration assays of *PTEN* WT (HCT116 *PTEN*^+/+^ and H1299) and *PTEN*-deficient (HCT116 *PTEN*^−/−^ and PC3) cells expressing vector only or vector encoding WT *PARK2* (HCT116 *PTEN*^+/+^, p = 8.1 × 10^−4^; HCT116 *PTEN*^−/−^, p = 0.99; H1299, p = 4.7 × 10^−3^; and PC3, p = 0.68). (F) BLI images of NOD/SCID mice retro-orbitally injected with luciferase-labeled H1299 cells expressing EV or human *PARK2*. Images were taken at 3 and 4 weeks post-injection (W3 and W4) to assess for luciferase-expressing lung metastases. (G) Quantification of BLI intensities on weeks 3 and 4 post-injection (EV, p = 0.02; and human *PARK2*, p = 0.54). The lungs were then dissected from the mice for H&E staining. (H) Scatterplot indicating that overexpression of *PARK2* significantly mitigated the formation of lung metastases in NOD/SCID mice (p = 0.006). (I) Representative H&E staining of lung sections 4 weeks post-injection to highlight significantly more and larger size metastatic tumor lesions in the mice injected with vector only or with vector encoding WT *PARK2* H1299 cells. Data are represented as mean ± SEM (n.s., not significant; ^∗^p < 0.05 and ^∗∗^p < 0.01, two-tailed t test). See also Figure S2.

To explore the significance of Akt activation in the molecular mechanisms that *PARK2* may contribute to tumor suppression, we tested the effect of *PARK2* overexpression in *PTEN* wild-type (WT) (HCT116 PTEN^+/+^ and H1299) and *PTEN* mutant (PC3 and HCT116 PTEN^−/−^) cell line models. Ectopic expression of *PARK2* led to the inhibition of Akt phosphorylation in HCT116 cells (Figure 3A). Importantly, we showed that *PARK2* overexpression led to the inhibition of Akt activation upon growth factor stimulation in *PTEN* WT H1299 cells (Figure 3B), but not in the PTEN mutant PC3 cells (Figure 3C). This reduction in Akt phosphorylation was also apparent when we ectopically expressed *PARK2* at levels near the endogenous Parkin levels expressed in H460 cells, for both the *EGFR* WT (H1299) and *EGFR* null (SW620) cells (Figures S2C and S2D). Unlike in parental PTEN^+/+^ HCT116 cells, overexpression of *PARK2* did not reduce Akt phosphorylation in isogenic PTEN^−/−^ HCT116 cells (Figure S2E), further supporting a role for *PTEN* in PARK2-mediated regulation of PI3K/Akt signaling.

Akt has been shown to play a central role in promoting growth factor-mediated cell proliferation and migration (Manning and Cantley, 2007). Given that *PARK2* inhibits Akt phosphorylation, we sought to investigate its functional contribution in suppressing some of the cellular functions mediated by Akt activation. HCT116 PTEN^+/+^ and H1299 cells exhibited a pronounced reduction in both cell proliferation (Figure 3D) and cell migration (Figure 3E) upon *PARK2* overexpression. However, this was not true for the *PTEN* mutant PC3 and isogenic PTEN^−/−^ HCT116 cells that showed no effect compared to control empty vector (EV)-expressing cells. No effect on cell proliferation and migration was also observed when overexpressing the E3-Ligase dead C431S Parkin mutant in both *PTEN* WT and mutant cells (Figures S2F–S2H).

To investigate the tumor suppressor effect of *PARK2* in vivo, we retro-orbitally injected luciferase-labeled H1299 cells overexpressing EV or human *PARK2*. Ectopic expression of *PARK2* significantly mitigated the formation of lung metastases (Figures 3F–3I) in NOD/SCID mice. The luciferase activity in cells expressing the *PARK2* gene was reduced on both weeks 3 and 4 post-injection (Figures 3F and 3G), and this was consistent with significantly lower number and smaller size of lung metastases, as histopathologically evaluated with H&E-stained sections (Figures 3H and 3I). These data show that suppression of Akt activation plays a central role in the tumor suppressor function of *PARK2* and that the mutational status of *PTEN* determines the functional contribution of *PARK2* loss in Akt-mediated cell proliferation and migration.

### *PARK2* Depletion Suppresses PTEN Protein Levels and Activity

**Figure 4:**
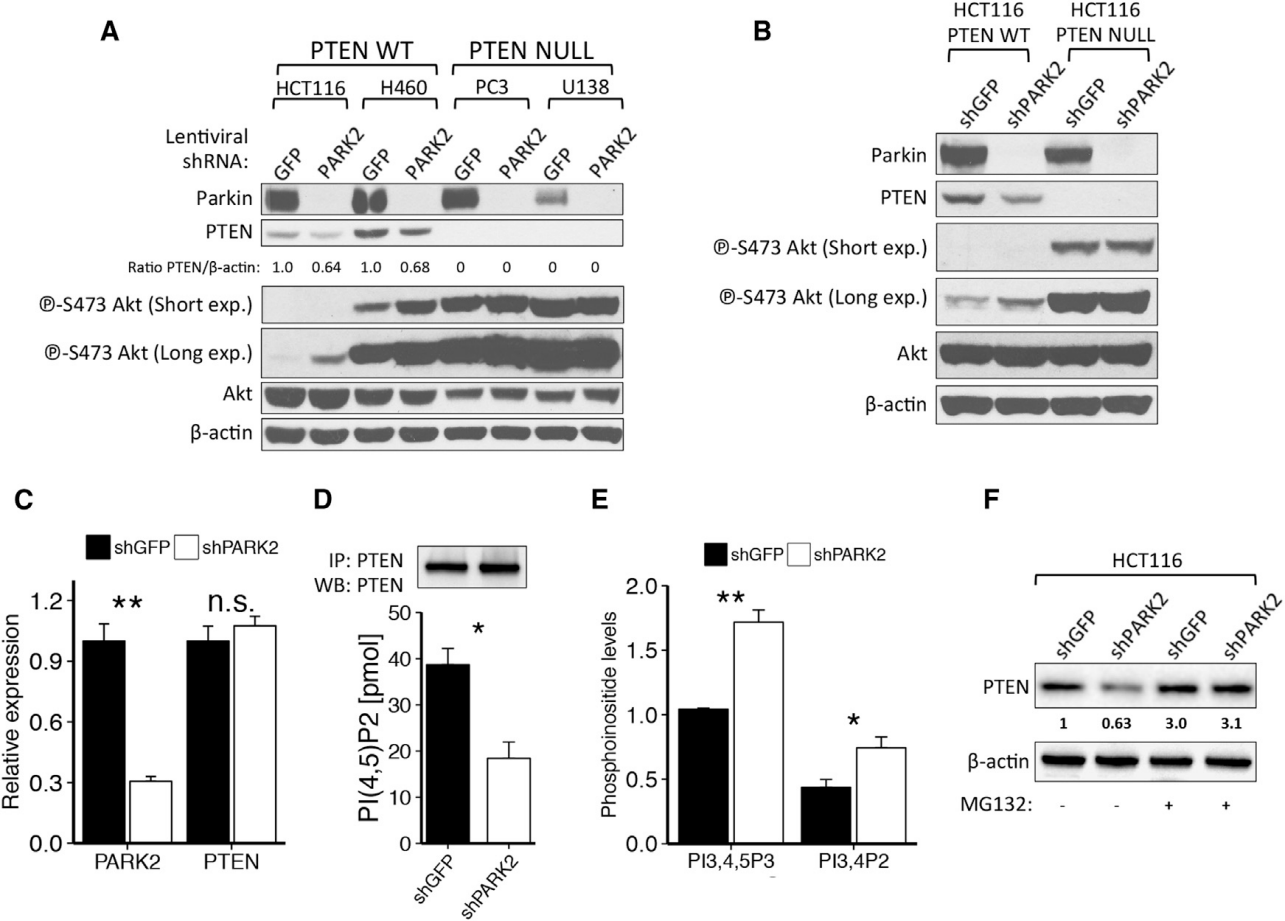
*PARK2* Depletion Contributes to Akt Activation in a *PTEN*-Dependent Manner (A and B) Immunoblotting of shGFP and shPARK2-expressing PTEN WT HCT116^parental^, H460 or *PTEN* mutant: PC3, U138 cells (A) and isogenic *PTEN* WT and NULL HCT116 cells (B). (C) Real-time qPCR for *PARK2* and *PTEN* on mRNA isolated from shGFP and shPARK2 HCT116 cells (*PARK2* qPCR, p = 0.001; and *PTEN* qPCR, p = 0.44). (D and E) Shown are the (D) PTEN activity assay (p = 0.015) and (E) phosphoinositide PI(3,4,5)P_3_ and PI(3,4)P_2_ levels between shGFP and shPARK2 HCT116 cells (PI(3,4,5)P_3_, p = 0.002; and PI(3,4)P_2_, p = 0.015). *PTEN* was ectopically expressed equally between cells. (F) Immunoblotting of shGFP and shPARK2 HCT116 cells with or without treatment with 10 μM MG132 for 6 hr. Data are represented as mean ± SEM (n.s., not significant; ^∗^p < 0.05 and ^∗∗^p < 0.01, two-tailed t test). See also Figures S3 and S4.

To further investigate the role of PTEN in *PARK2*-mediated regulation of the PI3K/Akt pathway, we performed *PARK2* knockdown analysis in cell lines with abundant Parkin but either WT *PTEN* present (HCT116 and H460) or absent (PC3 and U138). In line with our overexpression studies, *PARK2* knockdown induced Akt phosphorylation in HCT116 and H460 cells, but not in PC3 or U138 cells (Figures 4A, S3A, and S3B) or in the isogenic *PTEN* null HCT116 (Figure 4B) and MCF10A cells (Figure S3C). Most importantly, *PARK2* depletion led to a >30% reduction in PTEN protein levels across all *PTEN* WT cell lines (Figures 4A, 4B, and S3C), and this observation was confirmed in CRISPR/Cas9-mediated *PARK2* knockout H460 cells (Figure S3D). Previous studies with hypomorphic Pten allelic series of mice have shown that even subtle reductions in Pten protein levels can have dramatic consequences in cancer progression (Trotman et al., 2003). PTEN is regulated in cancer at the transcriptional, post-transcriptional, and post-translational levels (Song et al., 2012).

To gain further insight into this regulation by PARK2, we performed qPCR analysis of both *PARK2* and *PTEN* following lentiviral knockdown of *PARK2*, and we showed that *PARK2* depletion does not affect *PTEN* mRNA levels (Figures 4C and S3E). Importantly, *PARK2* depletion resulted in a significantly lower PTEN enzymatic activity (Figures 4D and S3F) and higher levels of PI(3,4,5)P_3_ and PI(3,4)P_2_ in cells (Figure 4E), further supporting the functional contribution of PTEN in PARK2-mediated regulation of the PI3K/Akt pathway. Moreover, we were struck to observe a reciprocal correlation between Parkin and PTEN protein expression across ten breast cancer cell lines that have previously been reported to be WT for *PTEN* (Figure S3G) (Saal et al., 2008).

In view of our findings, we speculated that suppression of PTEN protein levels by *PARK2* depletion might be regulated by proteasome-mediated protein degradation. Indeed, treatment with proteasome inhibitor MG132 led to an overall increase in PTEN protein levels and no significant difference between *GFP* and *PARK2* knockdown cells (Figures 4F and S3H). Taken together, these results show that *PARK2* depletion suppresses PTEN protein levels and activity, and they raise an interesting hypothesis that, mechanistically, this could be regulated, at least in part, by ubiquitin proteasome-mediated protein degradation.

### Increased Oxidative and Nitrosative Stress upon *PARK2* Depletion

**Figure 5:**
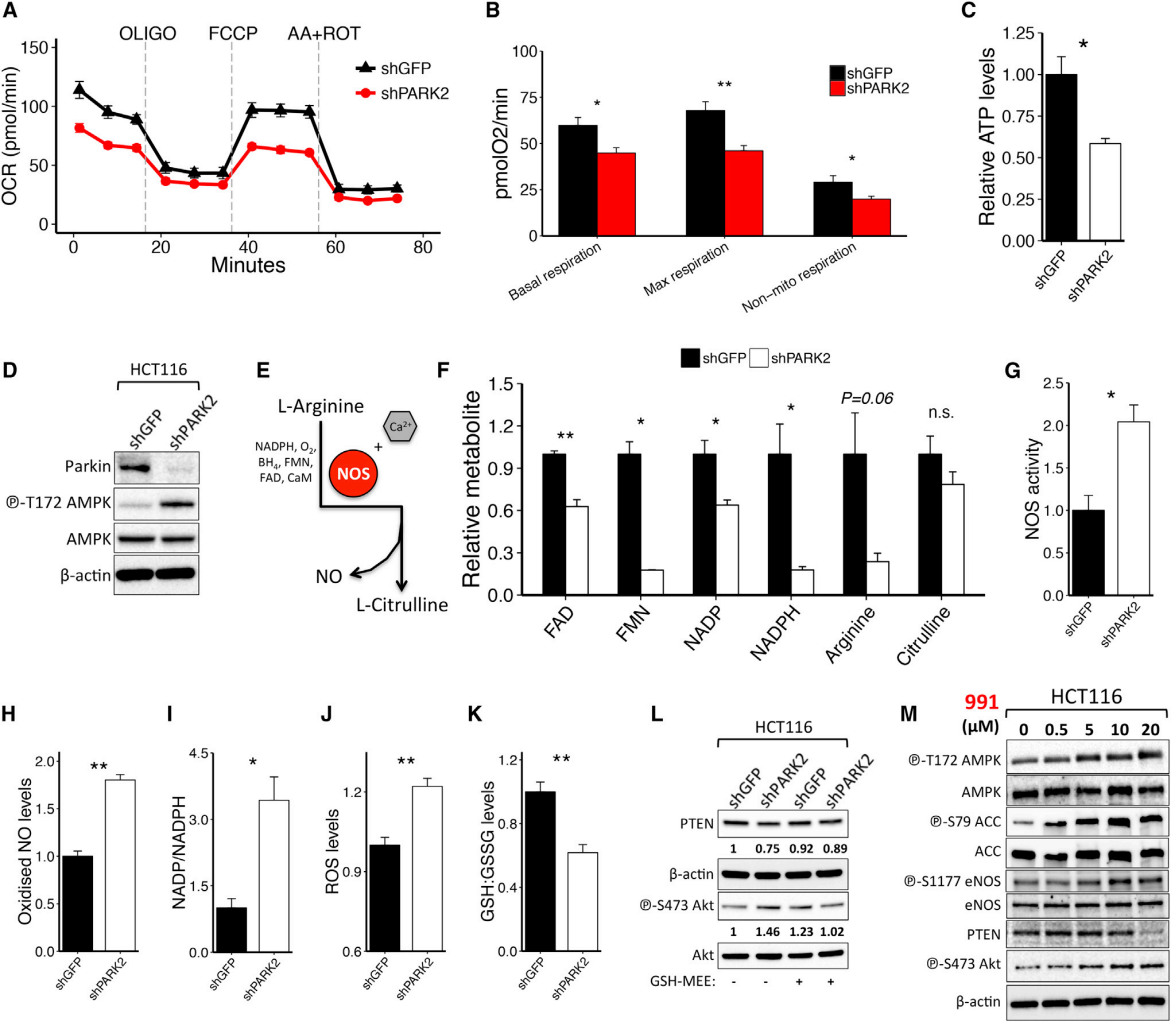
The Role of *PARK2* Depletion in Cellular Metabolism and AMPK-Mediated eNOS Activation (A–D) Shown are the (A) Seahorse analysis of OCR following sequential injection of oligomycin, carbonyl cyanide 4-(trifluoromethoxy) phenylhydrazone (FCCP), and antimycin A/rotenone (n = 6); (B) the OCR of basal (p = 0.016), maximal (p = 0.003), and non-mitochondrial respiration (p = 0.037); (C) the relative ATP levels (p = 0.02); and (D) immunoblotting analysis between shGFP and shPARK2 HCT116 cells. (E) Schematic representation of NO biosynthesis involving the conversion of L-arginine into L-citrulline by NOS in the presence of cofactors, including NADPH, FAD, FMN, CaM, O_2_, and BH_4_. (F) Relative abundance of metabolites involved in NO biosynthesis following *PARK2* knockdown in HCT116 cells (FAD, p = 0.002; FMN, p = 0.011; NADP, p = 0.025; NADPH, p = 0.019; Arginine, p = 0.063; and Citrulline, p = 0.24). (G–K) Shown are the (G) relative NOS activity (p = 0.017), (H) oxidized NO levels (p = 5.1 × 10^−4^), (I) NADP/NADPH levels (p = 0.013), (J) ROS levels following 2-hr menadione treatment (20 μM) (p = 0.006), and (K) GSH/GSSG levels between shGFP and shPARK2 HCT116 cells (p = 0.008). (L and M) Immunoblotting analysis of (L) shGFP and shPARK2 HCT116 cells, with or without treatment with 5 mM GSH-MEE for 72 hr, and (M) HCT116 cells following treatment with the indicated concentrations (0–20 μM) of the allosteric AMPK activator 991 for 5 hr. Cells were serum-starved for 2 hr prior to 991 treatment. Data are represented as mean ± SEM (n.s., not significant; ^∗^p < 0.05 and ^∗∗^p < 0.01, two-tailed t test). See also Figure S4.

Recent evidence shows that Parkin loss leads to a marked decrease in mitochondrial biogenesis (Stevens et al., 2015). To investigate the role of *PARK2* depletion in cellular metabolism, we initially measured the effect of knocking down *PARK2* expression on the oxygen consumption rate (OCR) of shGFP- and shPARK2-expressing cells, in the presence or absence of different mitochondrial stress inhibitors, using the Seahorse XFe96 Flux Analyzer. Notably, *PARK2* depletion resulted in a marked decrease in basal, maximal, mitochondrial, and non-mitochondrial respiration (Figures 5A, 5B, S4A, and S4B).

We next explored the effects of *PARK2* depletion on cellular metabolism by performing targeted metabolomic analysis. Consistent with lower OCR levels, *PARK2* knockdown cells showed a significant decrease in ATP levels (Figure 5C) and a concomitant activation of AMPK (Figures 5D and S4C), accompanied by the inhibition of mTORC1 signaling (Figures S4D and S4E). *PARK2* depletion also caused a reduction across many of the cofactors that contribute in nitric oxide (NO) biosynthesis, involving the conversion of L-arginine to L-citrulline (Figures 5E and 5F). Importantly, although all the anaplerotic substrates for NO synthesis were detected to be lower in the *PARK2* knockdown cells, L-citrulline levels were not significantly lower. Although other interpretations are possible, these results are consistent with a model in which NO synthase (NOS) is activated due to the loss of *PARK2*, such that the substrates for NOS are being consumed at a high rate while the product of NOS L-citrulline is maintained at a relatively constant level.

Indeed, consistent with this model, we measured the NOS activity and oxidized NO (NO_2_ + NO_3_) levels, and we found that they are significantly higher in *PARK2* knockdown cells (Figures 5G, 5H, and S4F). The latter also showed an overall increase in NADP/NADPH ratio (Figure 5I), higher reactive oxygen species (ROS) levels upon menadione treatment (Figures 5J and S4G), and lower reduced/oxidized glutathione (GSH/GSSG) levels (Figures 5K and S4H) compared to *GFP* control knockdown cells. Importantly, besides its function as a regulator of cellular redox status, GSH has a crucial role in modulating NO reactivity (Aquilano et al., 2014). NO as a free radical is a poor oxidant, hence, NO-dependent cysteine oxidation primarily occurs in the presence of oxygen or high ROS (Broniowska and Hogg, 2012). Of note, we showed that GSH-MEE (reduced ethyl ester) treatment restored the suppression in PTEN protein levels observed in *PARK2* knockdown cells (Figures 5L and S4I), while L-Buthionine-sulfoximine (BSO) treatment further enhanced the reduction in PTEN (Figure S4J). In addition, the activity of PTEN in *PARK2* knockdown cells in the presence of DTT was comparable to the shGFP cells without the DTT pre-incubation (Figure S4K), reinforcing the conclusion that the inhibition of PTEN is generated by a redox-dependent modification of cysteine residues.

Mitochondrial dysfunction and oxidative stress are well-recognized mechanisms leading to the activation of AMPK that can directly phosphorylate endothelial NOS (eNOS) to increase its catalytic activity (Schulz et al., 2009). In line with this, we showed that *PARK2* knockdown (Figure S4L) or allosteric activation of AMPK following treatment with the small molecule activator 991 (Figure 5M) led to an increase of eNOS phosphorylation and a reduction in PTEN protein levels. Of note, this reduction in PTEN protein levels and concomitant increase in Akt activation that were observed in 991-treated cells were decreased upon co-treatment with the NO scavenger carboxy-PTIO (cPTIO) (Figure S4M). Collectively, these results demonstrate that *PARK2* depletion leads to mitochondrial dysfunction, high eNOS activity that is mediated, at least in part, by AMPK activation, and high ROS levels that together coordinate efficient NO oxidation.

### *PARK2* Depletion Promotes PTEN S-Nitrosylation and Ubiquitination

**Figure 6:**
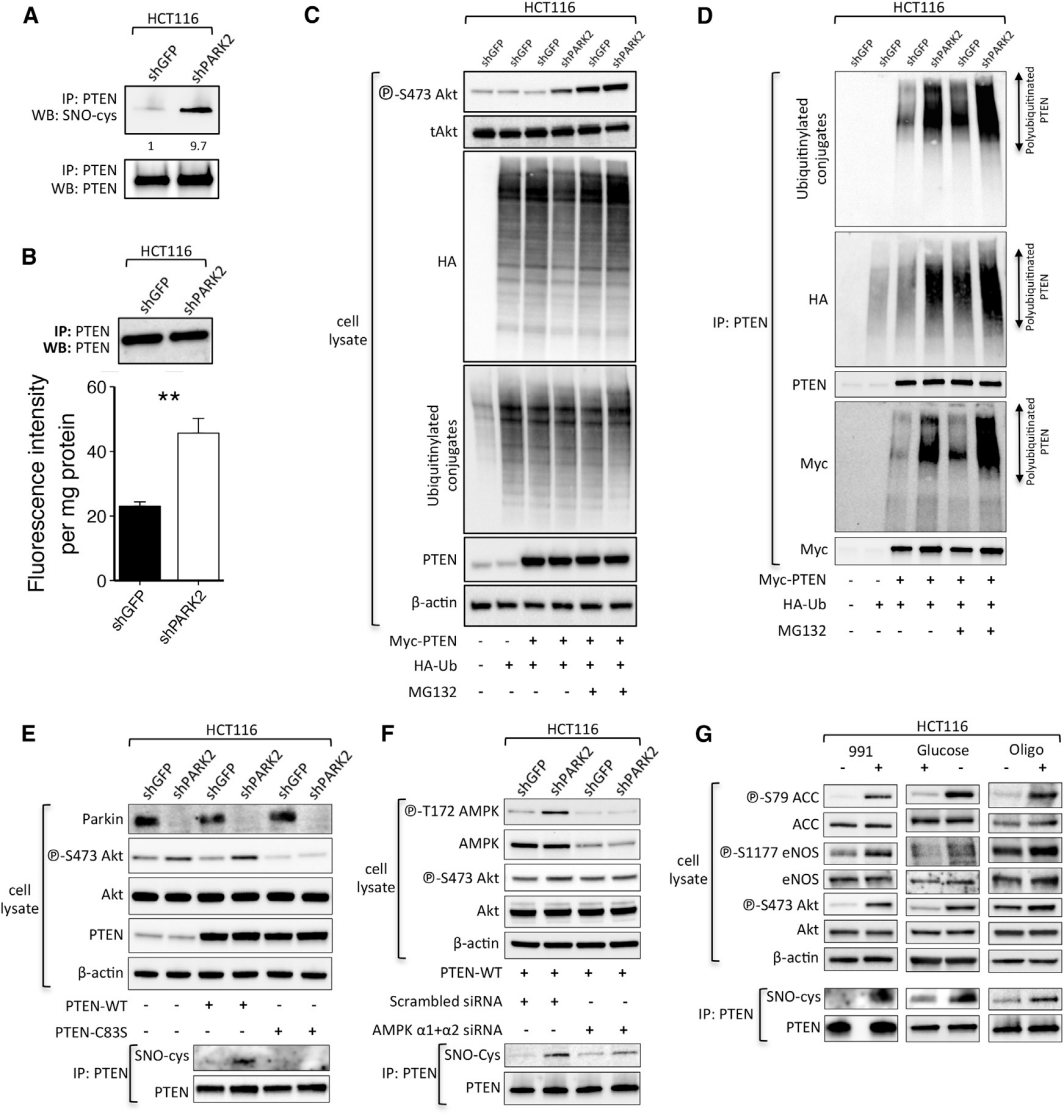
*PARK2* Depletion Leads to Enhanced S-nitrosylation and Ubiquitination of PTEN (A) Anti-PTEN immunoprecipitates (IP) derived from MYC-tagged-transfected *PTEN* HCT116 cells expressing *GFP* or *PARK2* shRNA. (B) Fluorometric measurement of S-nitrosylated PTEN between shGFP and shPARK2 HCT116 cells. SNO-PTEN was assessed by NO release, causing the conversion of DAN to the fluorescent compound NAT (p = 0.009). (C and D) Immunoblotting analysis of (C) whole-cell lysates and (D) anti-PTEN immunoprecipitates (IP) derived from HA-ubiquitin (Ub) and Myc-tagged *PTEN*-transfected HCT116 cells expressing *GFP* or *PARK2* shRNA. Where indicated, cells were treated with MG132 (10 μM) for 6 hr before collection. (E–G) Immunoblotting analysis and anti-PTEN immunoprecipitates derived from (E) Myc-tagged WT or C83S mutant *PTEN*-transfected HCT116 cells expressing *GFP* or *PARK2* shRNA; (F) WT *PTEN*-transfected shGFP and shPARK2 HCT116 cells, 48 hr post-transfection with scrambled or AMPK α1 and AMPK α2 siRNAs; and (G) parental HCT116 treated (or not treated) with the allosteric AMPK activator 991 for 5 hr (20 μM) following 2 hr serum starvation (left) or with 25-mM glucose-containing DMEM (middle) for 6 hr or with oligomycin (5 μM) for 2 hr. Data are represented as mean ± SEM. See also Figures S5 and S6.

High NO production exerts a pleiotropic range of biological functions that are regulated, in part, by a post-translational redox-mediated modification known as S-nitrosylation of protein cysteine residues. PTEN has previously been identified as a target of such modification (Kwak et al., 2010, Numajiri et al., 2011) that leads to the inhibition of its enzymatic activity and downstream activation of Akt signaling. In view of our previous findings, we speculated that *PARK2* depletion-mediated activation of the Akt pathway might be regulated by PTEN S-nitrosylation. Indeed, *PARK2* depletion resulted in a marked induction of PTEN S-nitrosylation (SNO-PTEN), as indicated both by immunoblotting immunoprecipitated PTEN with the anti-S-nitrosocysteine antibody (Figures 6A and S5B) and by measuring the release of NO from S-nitrosothiol of recombinant PTEN using the quantitative fluorescent 2,3-diaminonaphthalene (DAN) assay (Figures 6B and S5C). Interestingly, PTEN S-nitrosylation has previously been shown to promote its ubiquitination (Kwak et al., 2010), which could also explain the reduction in total PTEN protein levels and rescue following treatment with the proteasome inhibitor MG132 (Figures 4F and S3H). We performed an in-cell PTEN ubiquitination assay and showed that *PARK2* depletion resulted in a marked increase in PTEN ubiquitination, with or without pre-treatment with MG132 for 6 hr (Figures 6C, 6D, and S5D). Consistent with enhanced PTEN ubiquitination, we also found an increased abundance of conjugated ubiquitin (ub) on PTEN in *PARK2* knockdown HCT116 cells expressing HA-Ubiquitin (Figure S5A). An increase in phospho-Akt levels was observed in MG132-treated cells even in the absence of *PARK2* knockdown (Figure 6C), perhaps reflecting the increase in ub conjugates and PTEN ubiquitination (Figure 6D) resulting from proteasome inhibition.

Cysteine (Cys-83) has previously been identified as the critical cysteine thiol group within PTEN’s phosphatase domain that is predominantly targeted for S-nitrosylation, and, in line with this, C83S mutant PTEN is resistant to modification (Numajiri et al., 2011). Accordingly, we overexpressed WT and C83S mutant PTEN in the presence or absence of *PARK2* lentiviral small hairpin RNA (shRNA) to show that the C83S mutant was completely devoid of S-nitrosylation and rescued Akt activation in *PARK2* knockdown cells (Figure 6E). Importantly, since the activation of eNOS is also regulated by Akt-dependent phosphorylation (Dimmeler et al., 1999), our observation raised a dilemma as to whether the contribution of *PARK2* depletion to PTEN inhibition by S-nitrosylation is the consequence of Akt activation or is regulated via AMPK-mediated activation of eNOS independently of PI3K-Akt. To address this, we treated shGFP- and shPARK2-expressing HCT116 with the Akt inhibitor MK-2206 to show that inhibition of Akt activity does not rescue S-nitrosylation of PTEN (Figure S5E) nor the increased NO levels in *PARK2* knockdown cells (Figure S5F). On the contrary, small interfering RNA (siRNA)-mediated downregulation of *AMPK* (*AMPK* α1 and α2) led to a reduction in PTEN S-nitrosylation in *PARK2* knockdown cells (Figure 6F), further supporting the mechanistic implication of AMPK in PARK2-mediated activation of Akt.

To determine the importance of AMPK activation to trigger PTEN S-nitrosylation, we tested if AMPK activation alone is sufficient to induce this modification in the absence of *PARK2* depletion. Activation of AMPK following 991 treatment, glucose deprivation, or oligomycin treatment all led to a marked increase in PTEN S-nitrosylation (Figure 6G), identifying a functional cross-talk between AMPK and Akt activation.

To better understand the physiological importance of PTEN S-nitrosylation in the proliferation and survival of cells under conditions of metabolic stress, we performed clonogenic assays in *PTEN* null PC3 cells overexpressing EV, WT, or SNO-resistant (C83S) PTEN in the presence or absence of the glycolysis inhibitor 2-Deoxy-D-glucose (2-DG). Although no significant difference in colony formation was detected in PTEN WT-overexpressing cells with or without 2-DG treatment, there was a marked reduction (∼30%) in the number of colonies following 2-DG treatment in PC3 cells with enforced expression of the C83S mutant PTEN (Figures S6A–S6C). Moreover, we compared the half-maximal inhibitory concentrations (IC_50_s) of 2-DG and dichloroacetate (DCA) (PDK inhibitor), both of which result in the activation of AMPK in response to ATP depletion, to show that PC3 cells expressing the C83S mutant exhibited significantly higher sensitivity to treatment with these drugs, not evident in PTEN WT-expressing PC3 cells (Figures S6D and S6E). Last but not least, and consistent with the ability of *PARK2* knockdown cells to inactivate WT PTEN, we showed that shPARK2 cells proliferate significantly faster than shGFP-expressing cells in the presence of ectopic co-expression of WT (Figure S6F), but not C83S mutant, PTEN (Figure S6G). Taken together, these data demonstrate that *PARK2* depletion contributes to the activation of Akt signaling through promoting S-nitrosylation and ubiquitination of the tumor suppressor *PTEN*. Notably, our data also highlight a previously unexplored mechanism contributing to AMPK-mediated activation of PI3K/Akt involving the inhibition of PTEN by S-nitrosylation, which appears to be critical for the proliferative capacity and survival of PTEN-proficient cancer cells under conditions of energy stress.

### *Park2* Deletion Dramatically Promotes Tumorigenesis in *Pten* Heterozygous Knockout Mice

Given the functional contribution of *PARK2* depletion in PTEN inactivation by S-nitrosylation, we performed a bioinformatic analysis to report the copy number alterations (CNAs) of *PARK2* and *PTEN* across 995 cancer cell lines of the Cancer Cell Line Encyclopedia (CCLE) project. We were struck to identify that almost one-half of cell lines with HET deletion in *PTEN* (n = 145/314, 46.2%) also have HET deletion in *PARK2*, with the coexistence for loss of heterozygosity (LOH) at both the *PTEN* and *PARK2* loci being far more frequent than *PTEN* (n = 79/314, 25.2%) or *PARK2* (n = 119/344, 34.6%) LOH alone (Figure S7A). We extended this bioinformatic analysis summarizing the percentage of *PARK2* CNAs of primary tumors or cancer cell lines with *PTEN* LOH on 1,953 specimens across 13 different cancer types from the TCGA database to show that there is a strong selection for *PARK2* LOH, when one copy of the *PTEN* gene is missing (Figure S7B).

**Figure 7:**
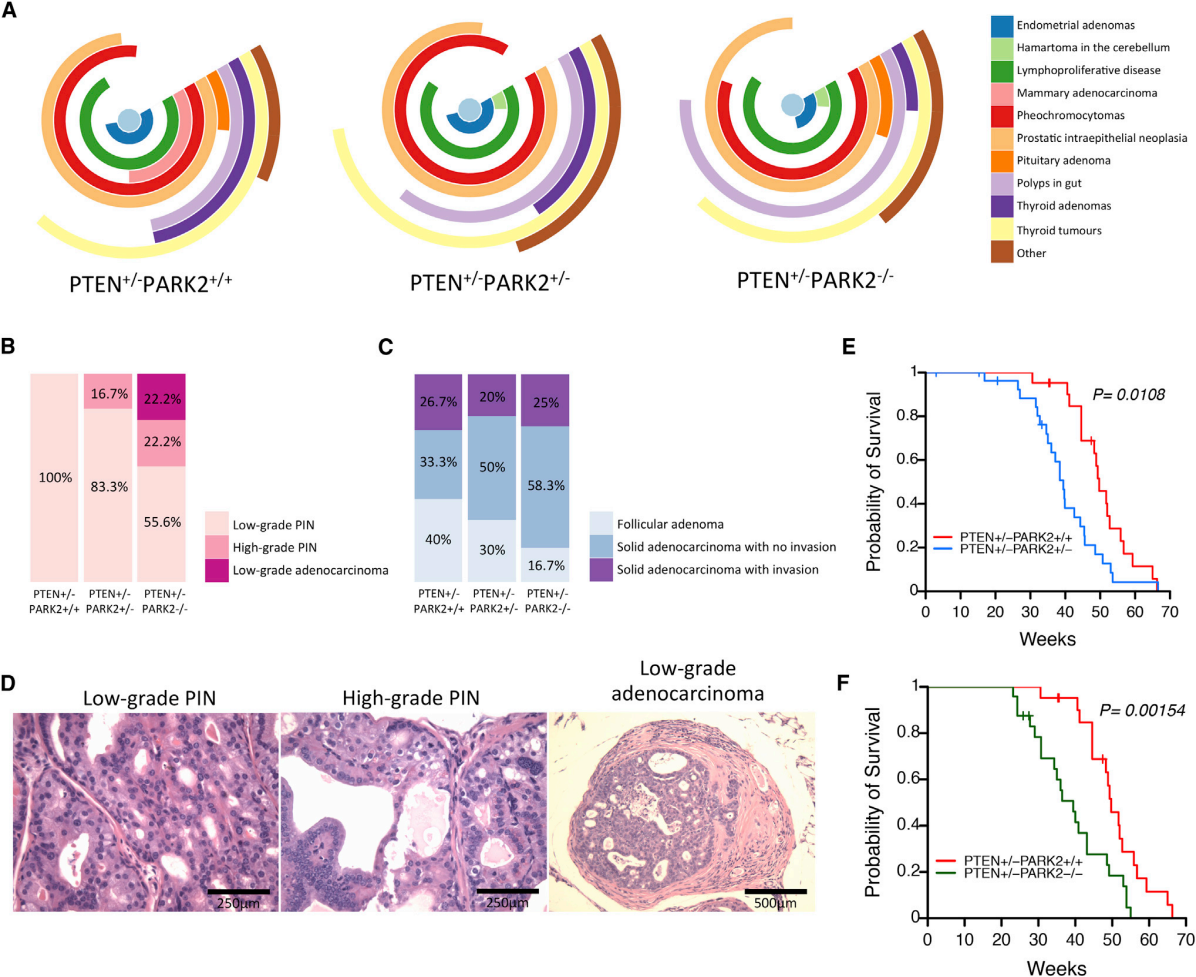
In Vivo Role of *Park2* Depletion in *Pten* Heterozygous Knockout Mice (A) Types and frequencies of hamartomas/adenomas/carcinomas/other neoplasms found in *Pten*^+/−^ mice with WT, HET, or HOM deletion of *Park2*. (B and C) Shown are the (B) frequencies of low- and high-grade prostatic intraepithelial neoplasia (PIN) lesions, prostatic low-grade adenocarcinoma, and (C) different stages of thyroid adenoma/carcinoma lesions found in *Pten*^+/−^ mice in the presence/absence of monoallelic or biallelic *Park2* deletion (prostate lesions, p = 9.3 × 10^−12^; and thyroid lesions, p = 0.002, chi-square test). (D) Representative H&E-stained sections of the low- (left) and high-grade (middle) PIN lesions and low-grade adenocarcinoma (right) found in *Pten*^+/−^ mice with or without *Park2* deletion. Scale bars, 250 (left and middle) and 500 μm (right). (E) Kaplan-Meier survival plot of *Pten*^+/−^ mice bearing WT (red) (n = 21) or HET (blue) (n = 27) *Park2* deletion (p = 0.0108, log-rank test). (F) Kaplan-Meier survival plot of *Pten*^+/−^ mice bearing WT (red) (n = 21) or HOM (green) (n = 24) *Park2* deletion. Both HET and HOM deletions of the *Park2* gene significantly increased the cancer-related mortality of *Pten*^+/−^ mice (p = 0.0015, log-rank test). See also Figure S7.

In an effort to study whether Park2 loss further exacerbates Pten-mediated tumorigenesis in vivo, mice with a targeted knockout of *Park2* exon 3 (Itier et al., 2003) were crossed with *Pten*^+/−^ mice (Di Cristofano et al., 1998). The latter were born viable and developed prostatic intraepithelial neoplasia (PIN), as well as numerous neoplastic lesions in many organs, including skin, colon, endometrium, liver, thyroid, and thymus (Di Cristofano et al., 1998, Podsypanina et al., 1999). Although *Pten*^+/−^/*Park2*^+/−^ and *Pten*^+/−^/*Park2*^−/−^ mice showed a similar spectrum of tumors like the ones detected in *Pten*^+/−^/*Park2*^+/+^ mice, they were significantly more tumor prone and exhibited features reminiscent of mice with a hypomorphic and a knockout Pten allele (Figure 7A). *Park2* loss was associated with an increased incidence of high-grade PIN and low-grade prostate adenocarcinoma (Figures 7B and 7D), as well as significantly higher numbers of solid thyroid adenocarcinomas (Figure 7C). Most importantly, mice bearing *Park2* loss developed tumor types, including hystiocytic sarcoma, multiple myeloma, and osteosarcoma in spine (Figures S7C and S7D), that have not been previously described for the *Pten*^+/−^ mice. On two occasions, mice bearing both *Park2* and *Pten* loss developed malignant pheochromocytomas and thyroid adenocarcinomas that metastasized to the lung (Figures S7C and S7D). As a result, *Pten*^+/−^/*Park2*^+/−^ and *Pten*^+/−^/*Park2*^−/−^ mice showed significantly poorer survival compared to *Pten*^+/−^/*Park2*^+/+^ mice (Figures 7E and 7F), while no difference in relative survival was found between *Pten*^+/−^/*Park2*^+/−^ and *Pten*^+/−^/*Park2*^−/−^ mice (Figure S7E).

To further assess the contribution of the proposed model on the *PARK2*-mediated inactivation of *PTEN* in vivo, we performed immunohistochemical (IHC) analysis of PTEN and phospho-Akt on tumor sections derived from *Pten*^+/−^ mice bearing WT, heterozygous (HET), or homozygous (HOM) deletion in *Park2*. Consistent with reduced PTEN and elevated phospho-Akt expression observed in the immunoblot analyses, *Pten*^+/−^ tumor epithelia bearing one or no copies of *Park2* expressed no or low PTEN protein levels, and they showed significantly higher phospho-Akt staining compared to lesions in *Pten*^+/−^/*Park2*^+/+^ mice (Figures S7F–S7H). Overall, our analysis showed that losses of function of Park2 and Pten displayed striking cooperativity to promote tumorigenesis and significantly shorten tumor-free survival.

## Discussion

The PI3K/Akt pathway represents a complex signaling network that integrates numerous upstream stimuli to regulate diverse cellular processes, including cell growth, proliferation, survival, and migration (Manning and Cantley, 2007). Interestingly, the *PARK2* gene is associated with the pathogenesis of disease states that are characterized by different responses in cell fate determination; therefore, understanding its functional contribution in the activation of survival pathways is clearly of immense clinical benefit.

In this study, we identify a PTEN-mediated role for *PARK2* depletion in the activation of the PI3K/Akt pathway. Of note, recent evidence suggested that PARK2 interacts with EGFR to promote its ubiquitination, thereby inhibiting Akt activation (Lin et al., 2015). However, the role of EGFR in PARK2-mediated activation of PI3K-Akt signaling might be more complex and/or cell type specific than previously appreciated, as ectopic PARK2 expression suppresses Akt activation even in *EGFR* null SW620 cells.

*PTEN* is a well-characterized haploinsufficient tumor suppressor (Di Cristofano et al., 1998), yet emerging evidence suggests that its function goes beyond its tumor suppressor role as a critical regulator of multiple CNS functions (Ismail et al., 2012). *PTEN* loss and subsequent activation of the PI3K/Akt signaling promotes the activation of mTOR signaling, which is hyperactive in many cancers (Carracedo and Pandolfi, 2008). Notably, the inhibition of mTORC1 by rapamycin treatment prevents PD symptoms in mice bearing a human mutation in the *PARK2* gene (Siddiqui et al., 2015). Furthermore, *PTEN* loss leads to the downregulation of PINK1 expression (Unoki and Nakamura, 2001), supporting a mechanism to suggest that *PARK2* LOH could repress PINK1 through inactivating PTEN and PINK1 repression could obliterate any residual activity of the remaining *PARK2* allele, by abrogating Parkin translocation to mitochondria.

An important mode of NO function primarily involves its reaction with oxygen or ROS and subsequent oxidation to nitrogen dioxide (NO_2_), dinitrogen trioxide (N_2_O_3_), or peroxynitrite (ONOO^−^), which ultimately lead to nitrosative stress and S-nitrosylation of target proteins and other biomolecules (Kovacs and Lindermayr, 2013). Of note, NO signaling and S-nitrosylation have previously been detected to contribute to PTEN inactivation in neurodegeneration (Kwak et al., 2010), but the role in cancer has been largely unknown. In this study, we showed that one of the most common genetic alterations across human cancers, *PARK2* loss, contributes to S-nitrosylation of PTEN, thereby promoting its ubiquitin-dependent degradation by the proteasome. We also report that there is a strong selection for co-occurrence of *PARK2* and *PTEN* LOH in cancer. This suggests that the high incidence of complete IHC loss of PTEN in *PTEN* HET tumors could be explained, at least in part, due to *PARK2* LOH, leading to S-nitrosylation and ubiquitination of the protein encoded from the remaining *PTEN* allele. In line with our previous observations (Poulogiannis et al., 2010), *PARK2* has many of the properties of a haploinsufficient tumor suppressor in that loss of a single *Park2* allele exacerbates tumorigenesis without requiring complete inactivation of the remaining allele.

Notably, activation of AMPK alone can trigger a marked increase in PTEN S-nitrosylation, pointing to a functional cross-talk between AMPK and Akt activation in the absence of any mTORC1-dependent negative feedback loop mechanisms (Efeyan and Sabatini, 2010). This redox-dependent modification in PTEN is important for supporting the survival and proliferative capacity of energy-deprived cancer cells, signifying an important compensatory role for AMPK to support cell homeostasis via PTEN-mediated activation of Akt signaling. Further studies are needed to determine the physiological functions of enhanced NO signaling across different disease states, but the reaction between NO and PTEN forms a nexus that opens up unique therapeutic opportunities for targeting dysregulated protein S-nitrosylation for a substantial fraction of tumors growing under conditions of energy deprivation.

## STAR★Methods

### Key Resources Table

**Table:** 

REAGENT or RESOURCE	SOURCE	IDENTIFIER
**Antibodies**		

Rabbit polyclonal anti-Akt	Cell Signaling Technology	Cat#9272; RPID: AB_329827
Rabbit monoclonal anti-phospho-Akt (Ser473)	Cell Signaling Technology	Cat#4060; RPID: AB_2315049
Rabbit monoclonal anti-GSK-3alpha/beta	Cell Signaling Technology	Cat#5676; RPID: AB_10547140
Rabbit monoclonal anti-phospho-GSK-3alpha/beta (Ser21/9)	Cell Signaling Technology	Cat#8566; RPID: AB_10860069
Rabbit monoclonal anti-PRAS40	Cell Signaling Technology	Cat#2691; RPID: AB_2225033
Rabbit monoclonal anti-phospho-PRAS40 (Thr246)	Cell Signaling Technology	Cat#2997; RPID: AB_2258110
Rabbit polyclonal anti-NDRG1	Cell Signaling Technology	Cat#5196; RPID: AB_10626626
Rabbit monoclonal anti-phospho-NDRG1 (Thr346)	Cell Signaling Technology	Cat#5482; RPID: AB_10693450
Rabbit monoclonal anti-p44/42 MAPK (Erk1/2)	Cell Signaling Technology	Cat#9102; RPID: AB_330744
Rabbit monoclonal anti-phospho-p44/42 MAPK (Erk1/2) (Thr202/Tyr204)	Cell Signaling Technology	Cat#9101; RPID: AB_331646
Rabbit polyclonal anti-4E-BP1	Cell Signaling Technology	Cat#9452; RPID: AB_10693791
Rabbit monoclonal anti-phospho-4E-BP1 (Thr37/Thr46)	Cell Signaling Technology	Cat#2855; RPID: AB_560835
Rabbit polyclonal anti-rabbit polyclonal anti-phospho-4E-BP1 (S65)	Cell Signaling Technology	Cat#9451; RPID: AB_330947
Mouse monoclonal anti-S6 Ribosomal Protein	Cell Signaling Technology	Cat#2317; RPID: AB_10694551
Rabbit polyclonal anti-phospho-S6 Ribosomal Protein (Ser235/236)	Cell Signaling Technology	Cat#2211; RPID: AB_331679
Rabbit monoclonal anti-phospho-S6 Ribosomal Protein (Ser240/244)	Cell Signaling Technology	Cat#5364; RPID: AB_10694233
Rabbit monoclonal anti-EGF Receptor	Cell Signaling Technology	Cat#4267; RPID: AB_2246311
Rabbit monoclonal anti-FoxO3a	Cell Signaling Technology	Cat#2497; RPID: AB_836876
Rabbit polyclonal anti-phospho-FoxO3a (Ser253)	Cell Signaling Technology	Cat#9466; RPID: AB_2106674
Rabbit polyclonal anti-HSP70	Cell Signaling Technology	Cat#4872; RPID: AB_10693928
Rabbit monoclonal anti-PTEN	Cell Signaling Technology	Cat#9559; RPID: AB_10695541
Rabbit polyclonal anti-AMPK-alpha	Cell Signaling Technology	Cat#2532; RPID: AB_330331
Rabbit monoclonal anti-phospho-AMPK-alpha (Thr172)	Cell Signaling Technology	Cat#2535; RPID: AB_331250
Rabbit monoclonal anti-Acetyl-CoA Carboxylase	Cell Signaling Technology	Cat#3676; RPID: AB_10694239
Rabbit monoclonal anti-phospho-Acetyl-CoA Carboxylase (Ser79)	Cell Signaling Technology	Cat#11818
Rabbit polyclonal anti-NOS (pan)	Cell Signaling Technology	Cat#2977; RPID: AB_2236063
Rabbit monoclonal anti-phospho-eNOS (Ser1177)	Cell Signaling Technology	Cat#9570; RPID: AB_823493
Mouse monoclonal anti-Myc epitope tag	Cell Signaling Technology	Cat#2276; RPID: AB_10693333
Mouse monoclonal anti-Parkin	Santa Cruz Biotechnology	Cat#sc-32282; RPID: AB_628104
Mouse monoclonal anti-PTEN	Santa Cruz Biotechnology	Cat#sc-7974; RPID: AB_628187
Mouse monoclonal anti-PTEN	Dako	Cat# M3627; RPID: AB_2174185
Mouse monoclonal anti-phospho-eNOS (Ser632)	Abcam	Cat#ab76199; RPID: AB_1523551
Mouse monoclonal anti-S-nitrosocysteine	Abcam	Cat#ab94930; RPID: AB_10697568
Mouse monoclonal anti-beta Actin	Abcam	Cat#ab6276; RPID: AB_2223210
Mouse monoclonal anti-mono- and polyubiquitinylated conjugates	Enzo Life Sciences	Cat#BML-PW8810; RPID: AB_10541840
Mouse monoclonal anti-HA.11 epitope tag	BioLegend	Cat#901501; RPID: AB_2565006
Rabbit polyclonal anti-Parkin	ThermoFisher	Cat#PA1-38412

**Chemicals, Peptides, and Recombinant Proteins**		

BKM120 (NVP-BKM120, Buparlisib) pan-PI3K inhibitor	Selleck Chemicals	Cat#S2247; CAS: 944396-07-0
BEZ235 (NVP-BEZ235, Dactolisib) dual PI3K and mTOR inhibitor	Selleck Chemicals	Cat#S1009; CAS: 915019-65-7
MK-2206 AKT inhibitor	Selleck Chemicals	Cat#S1078; CAS: 1032350-13-2
Rapamycin (Sirolimus) mTOR inhibitor	Selleck Chemicals	Cat#S1039; CAS: 53123-88-9
Trametinib (GSK1120212) MEK inhibitor	Selleck Chemicals	Cat#S2673; CAS: 871700-17-3
Torin 1 mTOR inhibitor	Tocris Bioscience	Cat#4247; CAS: 1222998-36-8
PD 0325901 MEK inhibitor	Tocris Bioscience	Cat#4192; CAS: 391210-10-9
Staurosporine	Sigma-Aldrich	Cat#S4400; CAS: 62996-74-1
Menadione	Sigma-Aldrich	Cat#M5625; CAS: 58-27-5
2-Deoxy-D-glucose (2-DG)	Sigma-Aldrich	Cat#D8375; CAS: 154-17-6
Sodium dichloroacetate (DCA)	Sigma-Aldrich	Cat#347795; CAS: 2156-56-1
Glutathione reduced ethyl ester	Sigma-Aldrich	Cat#G1404; CAS: 92614-59-0
L-Buthionine-sulfoximine	Sigma-Aldrich	Cat#B2515; CAS: 83730-53-4
Carboxy-PTIO	Sigma-Aldrich	Cat#C221; CAS: 148819-94-7
D-Glucose	Sigma-Aldrich	Cat#G8270; CAS: 50-99-7
Oligomycin	Sigma-Aldrich	Cat#75351; CAS: 579-13-5
MG-132	Sigma-Aldrich	Cat#M8699; CAS: 1211877-36-9
N-Ethylmaleimide	Sigma-Aldrich	Cat#E3876; CAS: 128-53-0
Protein G Sepharose, Fast Flow	Sigma-Aldrich	Cat#P3296
^3^H-myo-inositol	PerkinElmer	Cat#NET1168001MC
Puromycin	InvivoGen	Cat#ant-pr-5b
Blasticidin	InvivoGen	Cat#ant-bl-1
991 AMPK allosteric activator	Xiao et al., 2013	N/A
Sulforhodamine B (SRB)	Sigma-Aldrich	Cat#S1402; CAS: 3520-42-1

**Critical Commercial Assays**		

Seahorse XF Cell Mito Stress Kit	Agilent	Cat#103015-100
CellTiter96 AQueous non-radioactive cell proliferation Assay	Promega	Cat#G5421
Caspase-Glo 3/7 Assay	Promega	Cat#G8090
ROS-Glo H2O2 Assay	Promega	Cat#G8820
Nitric Oxide Assay Kit	Abcam	Cat#ab65327
GSH/GSSG-Glo Assay kit	Promega	Cat#V6611
Nitric Oxide Synthase (NOS) Activity Assay Kit	BioVision	Cat#K205-100

**Experimental Models: Cell Lines**		

HCT116 PTEN^+/+^ cells	Lee et al., 2004	N/A
HCT116 PTEN^−/−^ cells	Lee et al., 2004	N/A
MCF10A PTEN^+/+^ cells	Horizon	Cat#HD 101-006
MCF10A PTEN^−/−^ cells	Horizon	Cat#HD 101-006
JIMT-1 cells	DSMZ	Cat#ACC 589
HCT116 parental cells	ATCC	Cat#CCL-247
293T	ATCC	Cat#CRL-3216
PC-3	ATCC	Cat#CRL-1435
H460	ATCC	Cat#HTB-177
H1299	ATCC	Cat#CRL-5803
U-138 MG	ATCC	Cat#HTB-16
MDA-MB-134	ATCC	Cat#HTB-23
BT-474	ATCC	Cat#HTB-20
UACC-812	ATCC	Cat#CRL-1897
MCF7	ATCC	Cat#HTB-22
Hs 578T	ATCC	Cat#HTB-126
SK-BR-3	ATCC	Cat#HTB-30
AU565	ATCC	Cat#CRL-2351
BT-20	ATCC	Cat#HTB-19
ZR-75-30	ATCC	Cat#CRL-1504

**Experimental Models: Organisms/Strains**		

Mouse: C57BL/6J	Charles River	Stock No: 000664
Mouse: *Pten*^+/−^	Di Cristofano et al., 1998	N/A
Mouse: *Park2*^−/−^	Itier et al., 2003	N/A
Mouse: NOD.CB17-Prkdc^scid^/J	Charles River	Stock No: 001303

**Recombinant DNA**		

TRC Lentiviral eGFP shRNA positive control	Dharmacon	Cat#RHS4459
TRC Human PARK2 shRNA 1	Dharmacon	Cat# TRCN0000000283
TRC Human PARK2 shRNA 2	Dharmacon	Cat# TRCN0000000285
pLenti6/UbC/V5-DEST-empty vector	ThermoFisher	Cat#V49910
pLenti6/UbC/V5-DEST-PARK2-WT	This study	N/A
pLenti6/UbC/V5-DEST-PARK2-C431S	This study	N/A
pLV430G-oFL-T2A-eGFP	Green et al., 2015	N/A
pRK5-Myc-PTEN-WT	Song et al., 2011	N/A
pRK5-Myc-PTEN-C83S	This Study	N/A
HA-Ubiquitin	Addgene	Cat#18712
pHR-SIN-PTEN-WT	Addgene	Cat#30370
pHR-SIN-PTEN-C83S	This Study	N/A

**Sequence-Based Reagents**		

Silencer Select Non-targeting Negative Control	ThermoFisher	Cat#4390843
Silencer Select Pre-Designed siRNA against PRKAA1 (AMPKα1): siRNA ID: s100	ThermoFisher	Cat#4392420
Silencer Select Pre-Designed siRNA against PRKAA2 (AMPKα2): siRNA ID: s11056	ThermoFisher	Cat#4390824
LentiCRISPR v2	Addgene	Cat#52961
PARK2 sgRNA CRISPR/Cas9 All-in-One Lentivector (Human) (Target 3)	Applied Biological Materials	Cat#K1594608
Primers for human *PARK2* (F: GGTAGATCAATCTACAACAGCTTTTATG, R: TGCACTAGTCCCAGGGCA)	Hedrich et al., 2001	N/A
Primers for human *PTEN* (F: CAAGATGATGTTTGAAACTATTCCAATG, R: CCTTTAGCTGGCAGACCACAA)	Kim et al., 2004	N/A
Primers for human *18S* (F: GTGGAGCGATTTGTCTGGTT, R: CGCTGAGCCAGTCAGTGTAG)	Leonard et al., 2003	N/A

**Software and Algorithms**		

R statistical software (ver 3.2.3) and Bioconductor (ver 3.4)	The R Project	https://www.r-project.org/
		https://www.bioconductor.org/
Oncomine (ver 4.5)	ThermoFisher	https://www.oncomine.org/
GISTIC	Broad Institute	http://portals.broadinstitute.org/tcga/home
MultiQuant (ver 1.1)	SCIEX	https://sciex.com/products/software/multiquant-software
KM plotter	Szász et al., 2016	http://kmplot.com/analysis/

### Contact for Reagent and Resource Sharing

Further information and requests for resources and reagents should be directed to and will be fulfilled by the Lead Contact, George Poulogiannis (george.poulogiannis@icr.ac.uk).

### Experimental Model and Subject Details

#### Mice

*Pten*^+/−^ heterozygous mice (Di Cristofano et al., 1998) were bred with mice harboring a targeted knockout of *Park2* exon 3 (Itier et al., 2003). All mice were previously backcrossed over ten generations and maintained in a C57BL/6 background and they were genotyped by PCR for *Pten* and *Park2* alleles as described previously (Di Cristofano et al., 1998, Itier et al., 2003). Tumor-bearing mice were euthanized and subjected to whole-body histological analysis. Normal and tumor tissues were fixed in 4% PFA, embedded in paraffin, sectioned, and subjected to hematoxylin and eosin (H&E) staining for pathological evaluation. Survival analyses were performed using Kaplan–Meier curves and the Log-rank test. Six- to eight-week old NOD.CB17-Prkdc^scid^/J mice were injected via the retrobulbar sinus with 200 μl cell suspension (1x10^6^ cells) of GFP flow-sorted luciferase labeled (pLV430G-oFL-T2A-eGFP) H1299 cells expressing pLenti6∕UbC∕V5-DEST- empty vector or *PARK2*. Development of metastases was monitored by luciferin injection and bioluminescence imaging at 3 and 4 weeks post-injection. Bioluminescence (BLI) signal intensities were determined by using the region of interest (ROI) tool in Living Image Software (PerkinElmer). After the last imaging session the mice were euthanized and the lungs were surgically removed and inflated with 4% formalin in PBS. Lung sections, 5 μm, were subjected to H&E staining and the number of lung tumor nodules was counted using a dissection microscope. All mice were maintained according to NIH-approved institutional animal care guidelines and the study was approved by the Institutional Committee at the Beth-Israel Deaconess Medical Center.

#### Cell Culture

293T and PC3 cells were cultured in DMEM supplemented with 10% fetal bovine serum (FBS), 100 U/ml penicillin and 100 μg/ml streptomycin. HCT116 parental and isogenic *PTEN* null cell lines (Lee et al., 2004) were cultured in McCoy’s 5A, H460 and H1299 cells in RPMI, and U138 cells in DMEM-F12, all supplemented as above. The breast cancer cell lines MDA-MB-134, BT-474, UACC-812, MCF-7, Hs-578T, SK-BR-3, AU-565, BT-20 and JIMT-1 were cultured in DMEM, and the ZR-75-30 cells in RPMI, all supplemented as above. MCF10A parental and isogenic *PTEN* null cells were cultured in DMEM-F12 supplemented with 5% horse serum (HS) (ThermoFisher, 16050-122), 20 ng/ml EGF (PeproTech, AF-100-15), 0.5 mg/ml hydrocortisone (Sigma-Aldrich, H0888), 100 ng/ml cholera toxin (Sigma-Aldrich, C8052), 10 μg/ml insulin (Sigma-Aldrich, I1882), supplemented with penicillin and streptomycin as above. All cell lines were maintained at 37°C in a humidified incubator with 5% CO2 and were tested and confirmed to be negative for mycoplasma infection. For glucose starvation experiments, cells were washed twice with PBS and then incubated in DMEM without glucose and sodium pyruvate supplemented with 10% dialyzed FBS for 6 hr. For treatment with the allosteric AMPK activator 991 (Xiao et al., 2013), cells were serum-starved for 2 hr prior to 991 treatment for 5 hr. All cell lines used in the study were submitted to Eurofins Genomics for autosomal short tandem repeat (STR) authentication.

### Method Details

#### Transfections

For lentiviral gene knockdown, pLKO.1 shRNA sequences against human *PARK2* or *GFP* control were transfected in 293T cells using the FuGENE 6 transfection reagent (Promega, E2691) according to the manufacturer’s protocols. Infected cells were selected in the presence of 2 μg/ml puromycin (InvivoGen, ant-pr-5b) for at least 4 days. Stably expressing cancer cell lines were established by infecting with virus encoding human wild-type *PARK2*, the E3 ligase dead C431S mutant or empty vector in the pLenti6∕UbC∕V5-DEST backbone (ThermoFisher, V49910). Infected cells were selected in the presence of 2-10 μg/ml blasticidin (InvivoGen, ant-bl-1) for 2 weeks. For stable expression of luciferase and GFP, H1299 cells were infected with pLV430G-oFL-T2A-eGFP (Green et al., 2015), a lentivirus expressing the luciferase gene cloned in a eGFP expressing vector (gift from Dr Tina Yuan, Dana-Farber Cancer Institute, Harvard Medical School, Boston), and GFP-positive cells were sorted by flow cytometry 1 week after infection. H460 cells were transfected with *PARK2* sgRNA CRISPR/Cas9 All-in-One Lentivector Target 3 (Applied Biological Materials, K1594608) or control LentiCRISPR v2 according to the manufacturer’s instructions. Infected cells were selected in the presence of 2 μg/ml puromycin for 4 days and isolation of *PARK2* clonal deletion mutants was validated by immunoblotting analysis and DNA sequencing.

#### Immunoblot Analysis

Cells were washed with ice-cold PBS and lysed on ice for 30 min with cell lysis buffer containing 20 mM Tris-HCl (pH 7.5), 150 mM NaCl, 1 mM EDTA, 1 mM EGTA, 1% Triton X-100, supplemented freshly with a protease and phosphatase inhibitor cocktail (5872, Cell Signaling Technology), 10nM Calyculin A (Cell Signaling Technology, 9902) and 1 mM DTT (ThermoFisher, R0861). Lysates were subjected to centrifugation at 12,000 g for 10 min at 4°C and protein concentrations were determined using the Bradford assay (Bio-Rad, 5000006). Protein lysates were boiled for 10 min and subjected to SDS-PAGE electrophoresis. Densitometry was calculated using the Image Lab Software 5.2.1 (Bio-Rad).

#### Immunohistochemistry

The GBM tissue microarray (TMA) was prepared and characterized as described previously from the Tumor Tissue Bank at University Health Network, University of Toronto (Agnihotri et al., 2012). Briefly, the TMA was de-waxed in xylene followed by rehydration in a standard alcohol series. Antigen retrieval was by pressure cooking for 20 min in citrate buffer (pH 6.0), followed by blocking of endogenous peroxidase in 0.3% H_2_O_2_. Antibody incubation was performed using the ABC reagent kit (Vector Labs, PK-6100) as per manufacturers instructions. Briefly, Parkin antibody (ThermoFisher, PA1-38412) was diluted 1:100 in antibody diluent and added to the slides and incubated overnight at 4°C. Detection was performed using biotinylated secondary antibodies for 30 min, the ABC reagent kit and 3,3′-Diaminobenzidine chromogen. Sections were counter-stained with hematoxylin (ThermoFisher, 008001) for 30 s, dehydrated in 70, 80 and 100% ethanol, briefly washed in xylene and mounted in Permount (ThermoFisher, SP-15). For PTEN and phospho-AKT immunohistochemistry of mouse tumor sections, antigen retrieval was carried out by microwave heating for 20 min in citrate buffer (pH 6.0). Anti-PTEN (Dako, M3627) and anti-phospho-Akt (Ser473) (Cell Signaling Technology, 4060) antibodies were applied at dilutions of 1:200 and 1:50 respectively. PTEN and phospho-Akt IHC staining intensity was graded as 0 (negative), 1 (weak), 2 (moderate), and 3 (strong). Images were captured on a Nikon E-600 microscope and analyzed using Nikon ACT-1 software.

#### PTEN Phosphatase Activity Assay

For PTEN phosphatase activity assay, PTEN-transfected cells [pRK5-Myc-PTEN-WT, (Song et al., 2011)] were lysed in lysis buffer containing protease and phosphatase inhibitors in the absence of DTT. PTEN was immunoprecipitated from 1 mg of cell lysates and the PTEN phosphatase activity was measured using a PTEN activity ELISA kit (Echelon Biosciences Inc, K-4700), following the manufacturer’s instructions. Where indicated, PTEN immunoprecipitates were treated with 50 mM DTT at 4°C for 30 min prior to measuring PTEN phosphatase activity.

#### Detection of S-Nitrosylated PTEN

S-nitrosylation of PTEN was detected using the 2,3-diaminonaphthalene (DAN) assay. Briefly, PTEN transfected cells (pRK5-Myc-PTEN-WT) were lysed in lysis buffer containing protease and phosphatase inhibitors and 40 mM N-ethylmaleimide in the absence of DTT. PTEN was immunoprecipitated from equal volume of the diluted cell lysates containing 1 mg of soluble protein. The immunoprecipitates were washed twice with lysis buffer and twice with PBS. The pellet was resuspended in 500 μL of PBS and 100 μM HgCl_2_ and 100 μM DAN was added. The samples were incubated in the dark at room temperature (RT) for 30 min and 1 M NaOH was added. The generated fluorescent triazole from the reaction of DAN with the NO released from PTEN was measured using an excitation wavelength of 375 nm and an emission wavelength of 450 nm. As a negative control, the PTEN antibody alone in lysis buffer was immunoprecipitated and the resulting background fluorescence intensity was subtracted from each sample. S-nitrosylated PTEN was confirmed by immunoblotting of immunoprecipitated PTEN with anti-S-nitrosocysteine antibody (Abcam, ab94930), under non-reducing conditions. For non-reducing SDS-PAGE, β-mercaptoethanol was omitted from the loading buffer and samples were not boiled.

#### In Cell PTEN Ubiquitination Assay

Cellular assays to measure PTEN polyubiquitination were performed as described previously (Gupta et al., 2016). Briefly, cells were transfected with vectors encoding wild-type *PTEN* in the pRK5-Myc vector and HA-Ub and lysed in the presence of 40 NEM and supplemented freshly with a protease and phosphatase inhibitor cocktail. Equal volume of the diluted cell lysates containing 1mg of soluble protein was incubated with 1 μg mouse monoclonal anti-PTEN (Santa Cruz Biotechnology, sc-7974) or 2 μg mouse anti-HA (BioLegend, 901501) antibodies pre-coupled with protein G Sepharose beads (Sigma-Aldrich, P3296) and incubated at RT for 2 hr rotating. Beads were washed four times in immunoprecipitation buffer (50 mmol/L Tris, pH 7.6, 100 mmol/L NaCl, 2 mmol/L EDTA, and 0.2% Nonidet P-40) and the bound proteins were released by boiling in SDS-PAGE sample buffer for 10 min, prior to SDS-PAGE analysis and immunoblotting with the indicated antibodies.

#### ^3^H-Labeling of Phosphoinositides and HPLC Analysis

Subconfluent cells in 10-cm dishes were labeled in 8 mL of inositol-free DMEM and 10% dialyzed FBS supplemented with 160 μCi ^3^H-myo-inositol (PerkinElmer, NET1168001MC, specific activity = 20.1 Ci/mmol) for 24 hr. Deacylated phosphoinositides were resolved by HPLC using an Agilent 1200 Quaternary system, and radioactivity detected in-line using a Packard Flo-one Radiomatic detector. HPLC Buffer A is 1 mM EDTA, and Buffer B is 1 mM EDTA and 1 M NaH_2_PO_4_. An Agilent Zorbax SAX column (5 μm, 4.6 X 250 mm) was eluted by gradient program (from 100% A to 2% B at 1 min, 14% B at 30 min, 30% B at 31 min, 66% at 60 min, 100% B at 85 min, 100% A at 86 min, and hold at 100% A until 110 min) at a flow rate of 1 ml/min. PI(3,4)P_2_, PI(3,4,5)P_3_ standards were prepared by reacting PI3K with PI4P and PI5P, respectively and [γ-^32^P]ATP.

#### MTS and Caspase-3/7 Assays

Cell viability was assessed using the CellTiter96 AQueous non-radioactive cell proliferation assay [3-(4,5-dimethylthiazol-2-yl)-5-(3-carboxymethoxyphenyl)-2-(4-sulfophenyl)-2H-tetrazolium, inner salt; MTS] (Promega, G5421) according to the manufacturer’s protocol. Caspase-3/7 activities were measured using the Caspase-Glo 3/7 assay (Promega, G8090) following treatment with 50 or 100 nM staurosporine (Sigma-Aldrich, S4400) for 1 hr. For both cell viability and caspase 3/7 assays, HCT116 cells were seeded at 3x10^4^ cells/well in a 96-well plate and incubated overnight before treatment.

#### Cell Proliferation and Cell Migration Assays

Proliferation kinetics of H1299, PC3, HCT116 *PTEN*^+/+^ and HCT116 *PTEN*^−/−^ cells transfected with pLenti6/UbC/V5-DEST-PARK2-WT, PARK2-C431S mutant or empty vector were determined using the Sulforhodamine B (SRB) (Sigma-Aldrich, S1402) assay over a period of 5 days. The same method was applied for shGFP and shPARK2-expressing PC3 cells transfected with pHR-SIN-PTEN-WT, PTEN-C83S and control empty vector. Briefly, 2x10^3^ cells were seeded in a 96-well plate in triplicate and allowed to adhere for 24 hr (Day 0). Cells were fixed at days 0, 1, 3 and 5 as follows: they were fixed with 10% trichloracetic acid at 4°C for 60 min and washed 4x with 100 μL dH_2_O. After being left to dry at RT, the cells were stained for 10 min with 0.4% (w/v) SRB dissolved in 1% acetic acid, and protein-bound dye was extracted with 10 mM unbuffered Tris base pH 10.5. Absorbance was determined at 510 nm using a 96-well microplate reader and data presented indicate the average number of triplicate experiments ± standard error of the mean (SEM).

For cell migration assays, cells were starved overnight in 0.5% FBS and 5x10^4^ cells/well were seeded in 24-well transwell chambers (Corning, Lowell, MA, USA; 8 mm pore size) in a total volume of 300 μl 0.5% FBS-media. 750 μl of media (supplemented with 10%FBS) were added to the bottom of the transwells and the plates were returned to 37°C for 24 hr incubation. The transwell inserts were washed twice in PBS and cells were fixed in 10% trichloroacetic acid (TCA) for 1 hr. The TCA containing media was removed and the inserts were washed 3x in H_2_O. Cells were stained in 0.4% (w/v) SRB dissolved in 1% acetic acid for 10 min. The inserts were washed 3x in 1% acetic acid and the cells on the top of the filter were removed using a cotton swab. Migrated SRB-stained cells in the bottom of the transwell inserts were counted under a light microscope at 20x magnification in 5 fields/well.

#### Cytotoxicity and Clonogenic Assays

For determination of the IC_50_ values of 2-DG and DCA, shGFP- or shPARK2-expressing PC3 cells were co-transfected with pHR-SIN-PTEN-WT, PTEN-C83S and control empty vector. 48 hr post transfection, the cells were seeded at 5x10^3^ cells/well in a 96-well plate and treated with various concentrations of 2-DG (0.5-50 mM) or DCA (5-80 mM) for 72 hr followed by SRB staining. For clonogenic assays, cells were plated at 1 × 10^2^ cells/well in 24-well plates and treated with 10 mM DCA or 0.5 mM 2-DG. After 10 days, colonies were stained with a Crystal Violet solution (0.5% Crystal Violet, Sigma, 30% ethanol and 3% formaldehyde) and were counted in a GelCount™ (Oxford Optronix). Every experiment was performed in triplicate and data are presented as mean values ± SEM.

#### Real-Time qRT-PCR

Total RNA was extracted using the ReliaPrep RNA cell miniprep system (Promega, Z6010) according to the manufacturer’s instructions. Reverse transcription and real-time PCR reactions were carried out using the QuantiTect reverse transcription kit (QIAGEN, 205310) and SYBR select master mix (ThermoFisher, 4472908) respectively, using the TProfessional ThermoCycler from Biometra. The data presented are the mean values obtained ± SEM from triplicate reactions. Primer sequences used are as follows; Human *PARK2*, sense: TCAATCTACAACAGCTTTTATG, anti-sense: TGCACTAGTCCCAGGGCA (Hedrich et al., 2001), Human *PTEN*, sense: CAAGATGATGTTTGAAACTATTCCAATG, anti-sense: CCTTTAGCTGGCAGACCACAA (Kim et al., 2004), Human *18S*, sense: GTGGAGCGATTTGTCTGGTT, anti-sense: CGCTGAGCCAGTCAGTGTAG (Leonard et al., 2003).

#### ROS, NO, GSH/GSSG and NOS Activity Measurements

H_2_O_2_ levels were measured using the ROS-Glo H2O2 assay (Promega, G8820) according to the manufacturer’s instructions. Briefly, 1x10^4^ shGFP control and shPARK2-expressing cells were seeded in a 96-well plate and incubated at 37°C and 5% CO2 for 18 hr. Cells were washed with PBS and treated in Hank’s BSS medium containing 20 μM menadione (Sigma-Aldrich, M5625) in the presence of 20 μl 125 μM H_2_O_2_ substrate (provided in the kit). Following incubation at 37°C for 2 hr, 100 μl of ROS-Glo detection solution was added to the wells and the plate was incubated for 20 min at RT. Luminescence was determined with a microplate reader and the average relative light units (RLU) ± SEM of triplicate data are reported.

Nitric oxide (NO) levels were detected using the Nitric Oxide Assay Kit (abcam, ab65327) according to the manufacturer’s protocol. Briefly, shGFP and shPARK2-expressing cells were grown in regular culture medium for 24 hr. After washing with cold PBS, 2x10^6^ cells were resuspended in 500 μl assay buffer and homogenized quickly by pipetting up and down a few times. The samples were centrifuged for 5 min at 4°C at top speed to remove any insoluble material. Triplicate 75 μl aliquots of the supernatant were transferred to a 96-well plate and 5 μl of enzyme cofactor and 5 μl of nitrate reductase were added to each of the reaction wells. The plate was incubated at RT for 3 hr and 5 μl of enhancer was added to each well, before further incubation for 30 min at RT. 5 μl of the DAN probe was added to each well, the plate was incubated at RT for 10 min and 5 μl of NaOH was added before final incubation at RT for 10 min. Fluorescence was determined with a microplate reader at Ex/Em = 360/450nm. All reagents were provided in the kit. GSH:GSSG levels were measured using the GSH/GSSG-Glo Assay kit (Promega, V6611) and nitric oxide synthase (NOS) activity was determined using the NOS activity assay kit (BioVision, K205-100) according to the manufacturer’s protocol.

#### Mitochondrial Flux Analysis

*PARK2* and *GFP* stable knockdown cells were plated at 2x10^4^/well in a 96-well Seahorse cell culture microplate and incubated overnight. The next morning, culture media was replaced with pH-adjusted (pH = 7.4 ± 0.1) bicarbonate-free DMEM with 10 mM Glucose, 1 mM sodium pyruvate and 2 mM L-Glutamine and the plate was incubated at 37°C for 1 hr in a non–CO2 incubator. Oxygen consumption rates were measured using the Seahorse XF Cell Mito Stress Kit (Agilent, 103015-100) on a XFe96 Analyzer. 2 μM Oligomycin, 0.5 μM FCCP, and 0.5 μM rotenone/antimycin (R/A) were used for all conditions. Cell numbers were normalized using Cyquant (ThermoFisher, C35012).

#### Tandem Mass Spectrometry

Metabolite levels were determined by targeted liquid-chromatography tandem mass spectrometry (LC-MS/MS) analysis as described previously (Yuan et al., 2012). Briefly, 48 hr prior to each experiment, 2.5x10^5^ HCT116 cells expressing shGFP or shPARK2 were seeded in 6-cm dishes. The media for each plate was replaced 2 hr prior to metabolite extraction and after aspiration, 4 mL pre-chilled (at −80°C) methanol was added to the cells on dry ice for 15 min. Cell extracts were collected into 15 mL conical tubes and centrifuged for 5 min at 4200 rpm. Solvent in the resulting supernatant was evaporated using a centrifugal vacuum evaporator (“SpeedVac”) and samples were re-suspended in 20 μL HPLC-grade water for mass spectrometry. 8 μL were injected and analyzed using a Prominence UFLC HPLC system (Shimadzu) for hydrophilic interaction liquid chromatography (HILIC), coupled to a QTRAP 5500 hybrid triple quadrupole/linear ion trap mass spectrometer (SCIEX) operated in the selected reaction monitoring (SRM) mode. Peak areas of LC-SRM-MS traces for each metabolite were integrated using the MultiQuant v1.1 software (SCIEX).

### Quantification and Statistical Analysis

#### Bioinformatic Analysis

GISTIC (Genomic Identification of Significant Targets in Cancer) analyses were performed on DNA copy number data from the SNP pipeline version 3.0. 9,863 tumor samples across 28 cancer types were selected for this analysis using the 2015-06-01 stddata TCGA/GDAC tumor sample sets from FireHose, and the frequencies of *PARK2* DNA copy number changes were plotted using a polar histogram. The Oncomine cancer profiling database was used to analyze *PARK2* mRNA expression across 13,499 cancerous and 2,708 normal tissue specimens from 125 independent microarray datasets representing 27 different cancer types. The degree of *PARK2* mRNA underexpression (≥0.5 and ≥ 1-log2-fold change in tumor versus normal) was plotted across all tumor types. Kaplan-meier analysis in Figure S1C was performed using the KM plotter (Szász et al., 2016). Student’s t test and one- or two-way ANOVA were used to evaluate the statistical significance among different variables as indicated in the respective figure legends; n.s. (not significant), ^∗^ (p < 0.05), ^∗∗^ (p < 0.01).

## Author Contributions

G.P. and L.C.C. designed the study and wrote the manuscript. G.P., A.G., S.A.-V., N.K., M.D., A.V., Y.Z., Y.-H.C., and D.A. performed molecular biology experiments. S.A. and G.Z. performed Parkin IHC in GBM tumors. M.J.A. analyzed histopathology data. G.P. and J.M.A. performed metabolomics and analyzed the data. G.P. and R.C.-W. performed retro-orbital injection experiments and contributed to mouse colony management. G.P. performed bioinformatic analysis. All authors commented on the manuscript.

## Supplemental Information

Document S1. Figures S1–S7

Table S1. Number and Type of Tumors Used to Report the Frequency of PARK2 DNA Copy Number Loss from the TCGA Dataset, Related to Figure 1

Table S2. Number and Type of Tumors that Showed Significantly Lower PARK2 mRNA Expression Compared to Their Normal Counterparts, Related to Figure 1

Document S2. Article plus Supplemental Information
