# Supplementary material for: PARK2 Depletion Connects Energy and Oxidative Stress to PI3K/Akt Activation via PTEN S-Nitrosylation
Source: Mol Cell. 2017 Mar 16;65(6):999–1013.e7. doi: 10.1016/j.molcel.2017.02.019 (PMC5426642; doi:10.1016/j.molcel.2017.02.019)
Supplement: Document S1. Figures S1–S7 [file mmc1.pdf]

Molecular Cell, Volume *65*

Supplemental Information

PARK2 Depletion Connects Energy and Oxidative Stress to PI3K/Akt Activation via PTEN S-Nitrosylation

Amit Gupta, Sara Anjomani-Virmouni, Nikos Koundouros, Maria Dimitriadi, Rayman Choo-Wing, Adamo Valle, Yuxiang Zheng, Yu-Hsin Chiu, Sameer Agnihotri, Gelareh Zadeh, John M. Asara, Dimitrios Anastasiou, Mark J. Arends, Lewis C. Cantley, and George Poulogiannis

SUPPLEMENTAL FIGURES

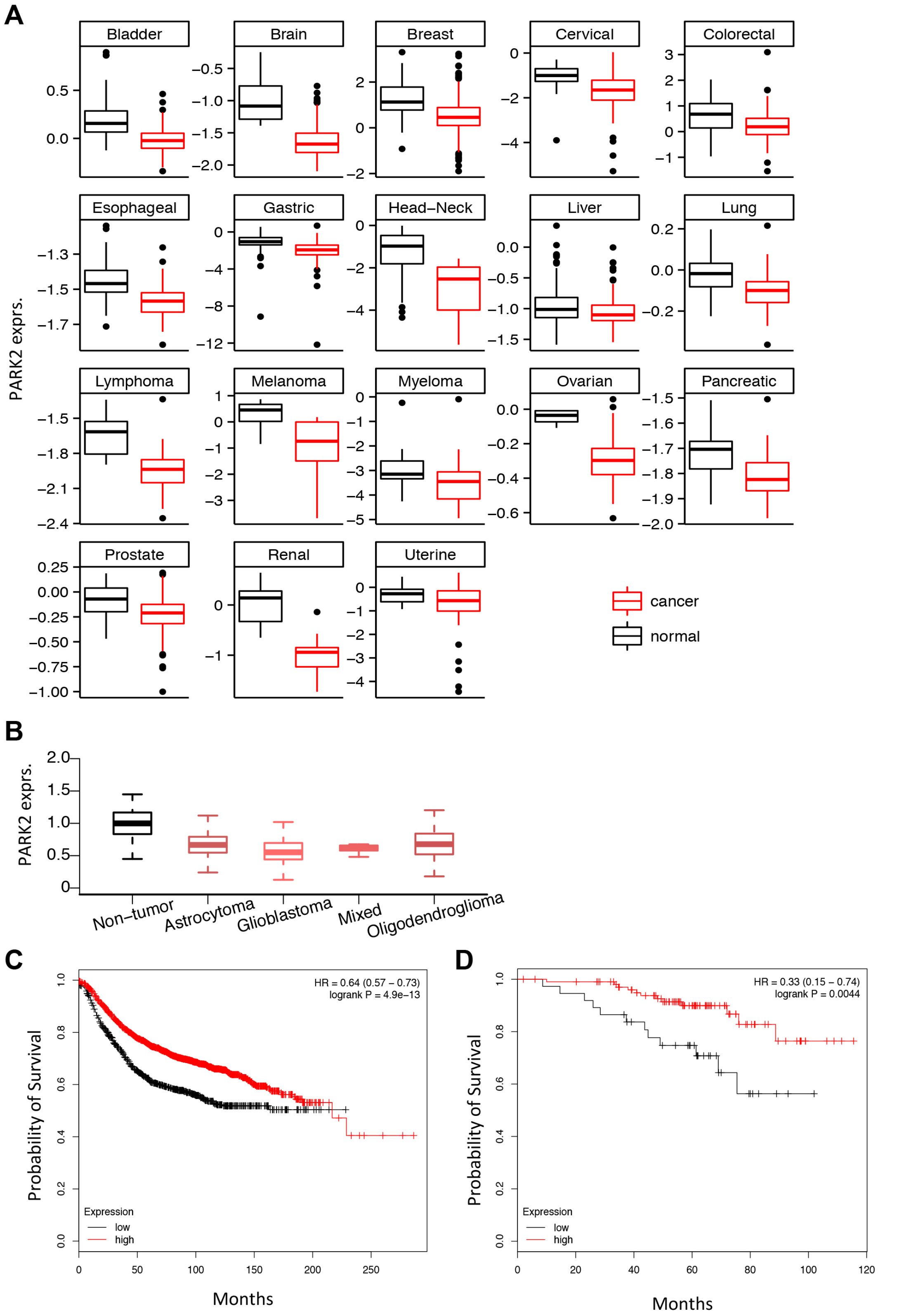

Figure S1, related to Figure 1. *PARK2* is significantly under-expressed across many tumor types and its loss is associated with poorer prognosis.

(A) Representative box-plots indicating significantly lower *PARK2* mRNA expression in cancerous versus corresponding normal tissue across many different tumor types including bladder; $p = 5.184 \times 10^{-11}$, brain; $p = 0.0003$, breast; $p = 3.103 \times 10^{-10}$, cervical; $p = 0.001$,

colorectal; $p = 0.016$, esophageal; $p = 1.145 \times 10^{-6}$, gastric; $p = 1.832 \times 10^{-5}$, head and neck; $p = 4.44 \times 10^{-5}$, liver; $p = 7.052 \times 10^{-5}$, lung; $p = 1.607 \times 10^{-6}$, lymphoma; $p = 1.251 \times 10^{-7}$, melanoma; $p = 0.036$, myeloma; $p = 0.015$, ovarian; $p = 0.0003$, pancreatic; $p = 0.005$, prostate; $p = 0.0003$, renal; $p = 4.803 \times 10^{-5}$, uterine; $p = 0.005$ (Oncomine database). 2-tailed *t*-test was used to evaluate differential expression of *PARK2* between cancer and normal tissues.

(B) *PARK2* mRNA expression across non-tumor and different histological subtypes of glioma specimens from the REpository for Molecular BRAin Neoplasia DaTa (REMBRANDT) ($p = 0.0007$, one-way Anova analysis).

(C) Kaplan-meier survival plots of breast cancer patients stratified by tumors bearing *PARK2* low (n=1786, defined as below the 25th percentile) versus high (n=5424, defined as above the 25th percentile) mRNA expression and (D) lung adenocarcinomas with *PARK2* low (n=127) versus high (n=376) mRNA expression. Log-rank test was used to compare the respective survival curves (from left to right: $p = 4.9 \times 10^{-13}$, $p = 0.0044$). This analysis was performed using the KM plotter.

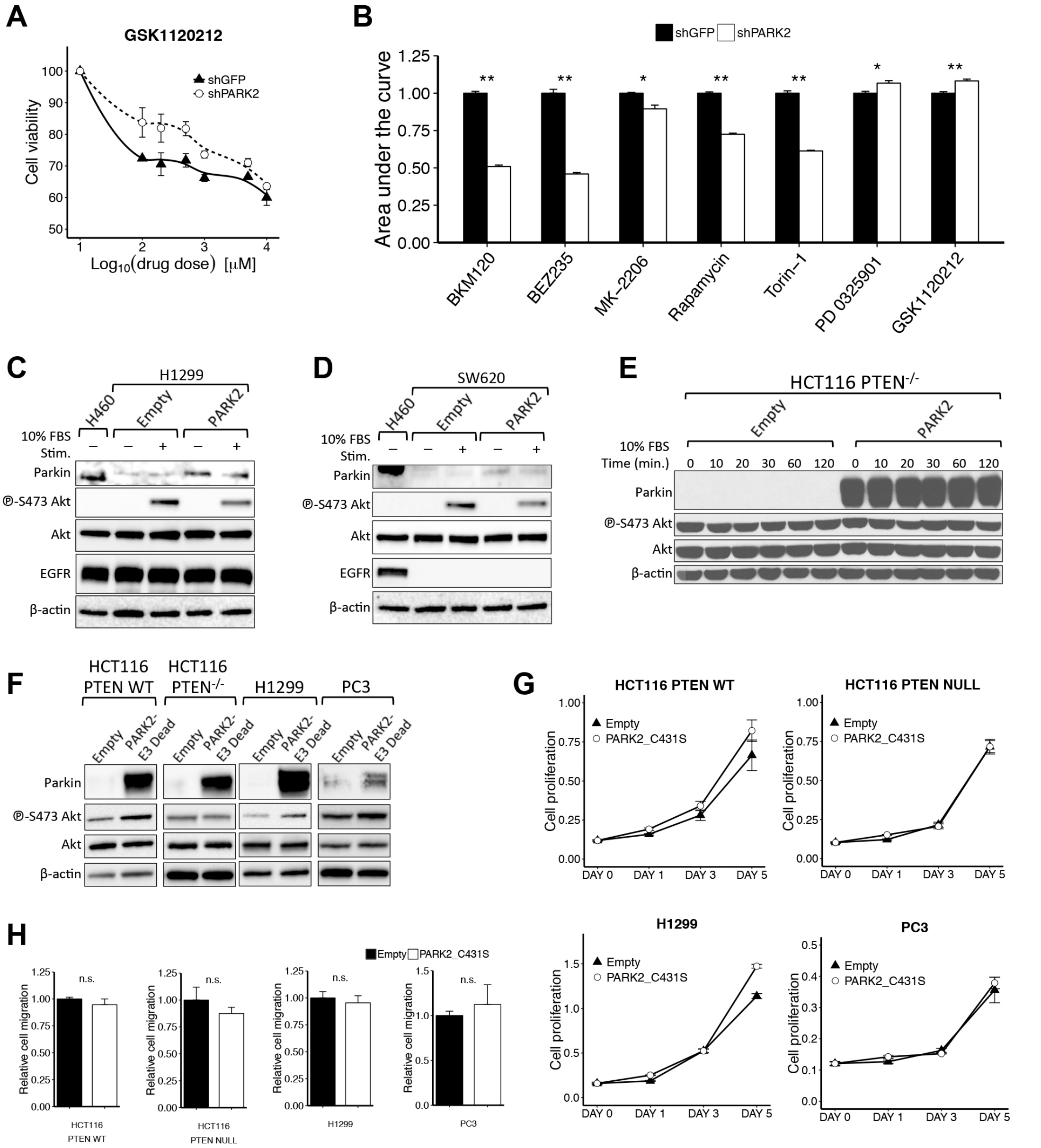

Figure S2, related to Figures 2 and 3. Tumor suppressor function of *PARK2* through regulation of PTEN-mediated Akt activation.

(A) Drug-response curve indicating that *PARK2* knockdown significantly mitigates the response to MEK inhibitor (GSK112012) (p = 0.03, two-way Anova analysis).

(B) Area under the curve analysis of the dose-response curves presented in Figures 2E and 2SA indicating that *PARK2* knockdown selectively sensitizes HCT116 cells to inhibitors of the PI3K/Akt/mTOR, but not the MAPK pathway.

Immunoblotting analysis of

(C) H1299 (*EGFR* wild-type) and (D) SW620 (*EGFR* null) cells with ectopic Parkin expression close to the endogenous levels in H460 cells. Cells were subjected to 24 hour serum starvation before stimulation with 10% FBS for 10 minutes.

(E) EV and *PARK2*-overexpressing *PTEN* null HCT116 cells following 10% FBS stimulation. Cells were serum-starved for 24 hours prior to FBS stimulation,

(F) *PTEN*-wild-type (HCT116 $PTEN^{+/+}$ and H1299) and *PTEN*-deficient (HCT116 $PTEN^{-/-}$ and PC3) cells expressing EV or the E3 ligase-dead Parkin C431S mutant.

(G) Cell proliferation and (H) Cell migration assays of *PTEN*-wild-type and *PTEN*-deficient cells expressing EV or Parkin C431S. Statistical significance between proliferation curves was assessed using two-way ANOVA analysis (HCT116 $PTEN^{+/+}$; p = 0.169, HCT116 $PTEN^{-/-}$; p = 0.947, H1299; p = 0.919, PC3; p = 0.751), and between migration assays using 2-tailed

t-test (HCT116 *PTEN*$^{+/+}$; $p = 0.392$, HCT116 *PTEN*$^{-/-}$; $p = 0.403$, H1299; $p = 0.633$, PC3; $p = 0.617$). Data are represented as mean ±SEM (n.s.: not significant, *$p < 0.05$, **$p < 0.01$).

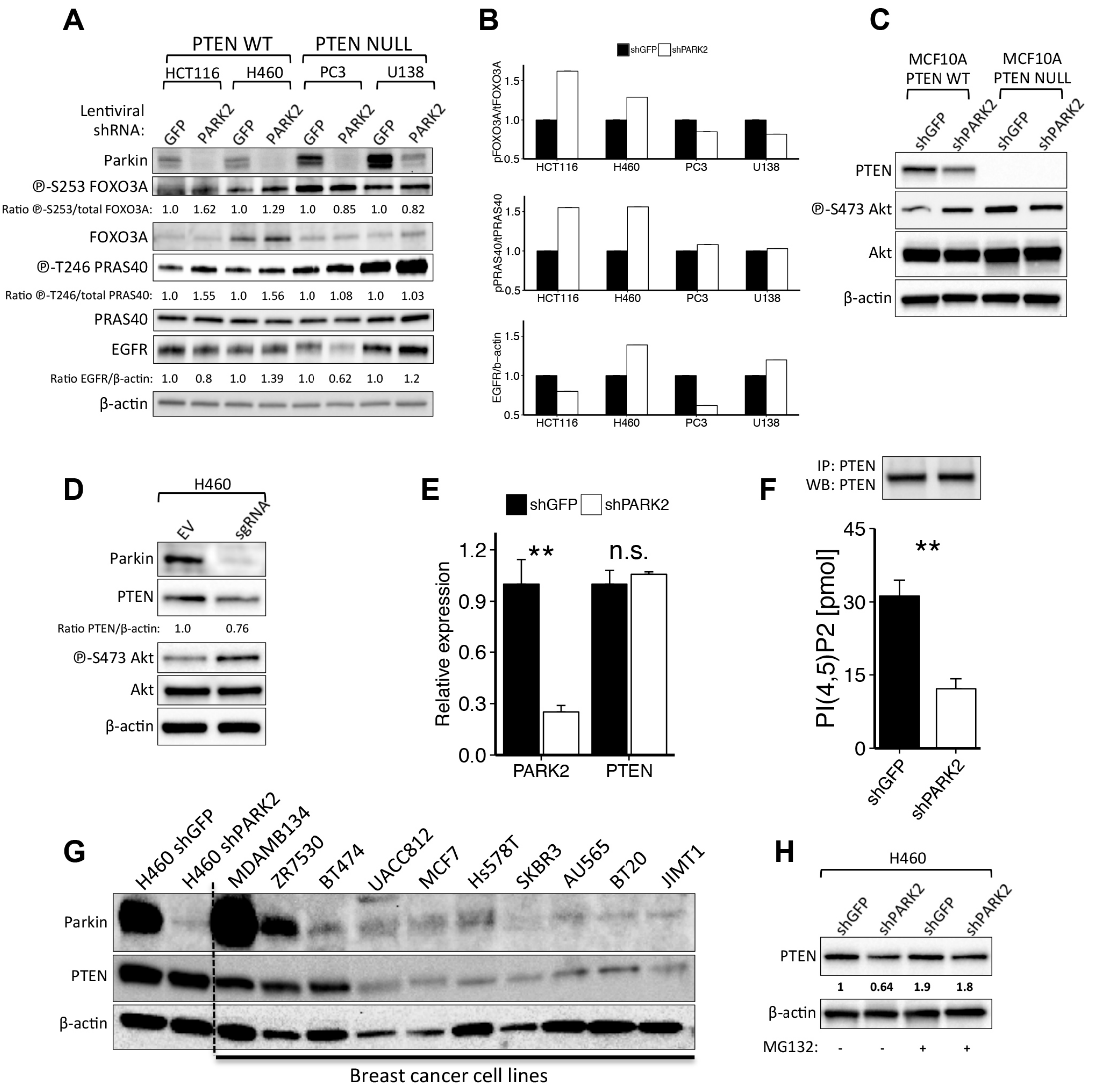

Figure S3, related to Figure 4. The role of *PARK2* depletion in PTEN-mediated Akt activation.

(A) Immunoblotting analysis and (B) corresponding densitometry plot of *PTEN* WT and deficient cell lines for PI3K/Akt activation markers.

Immunoblotting analysis of

(C) H460 cells transfected with control or CRISPR/Cas9-plasmids targeting the *PARK2* gene and

(D) MCF10A *PTEN* WT and isogenic null cell lines,

(E) Quantitative real-time PCR for *PARK2* and *PTEN* (*PARK2*; p = 0.007, *PTEN*; p = 0.52) and

(F) PTEN activity assay between shGFP and shPARK2 H460 cells (p = 0.008, 2-tailed *t*-test). PTEN was ectopically expressed equally between shGFP and shPARK2 H460 cells.

Immunoblotting analysis of

(G) A panel of breast cancer cell lines (n=10) showing a statistically significant reciprocal correlation between Parkin and PTEN protein expression following densitometry analysis (Correlation coefficient: 0.79, p = 0.007, Pearson's correlation). shGFP and shPARK2 H460 cells were used as control for Parkin expression.

(H) shGFP and shPARK2 H460 cells with or without treatment with 10μM MG132 for 6 hours. Data are represented as mean ±SEM (n.s.: not significant, *p < 0.05, **p < 0.01, 2-tailed *t* test).

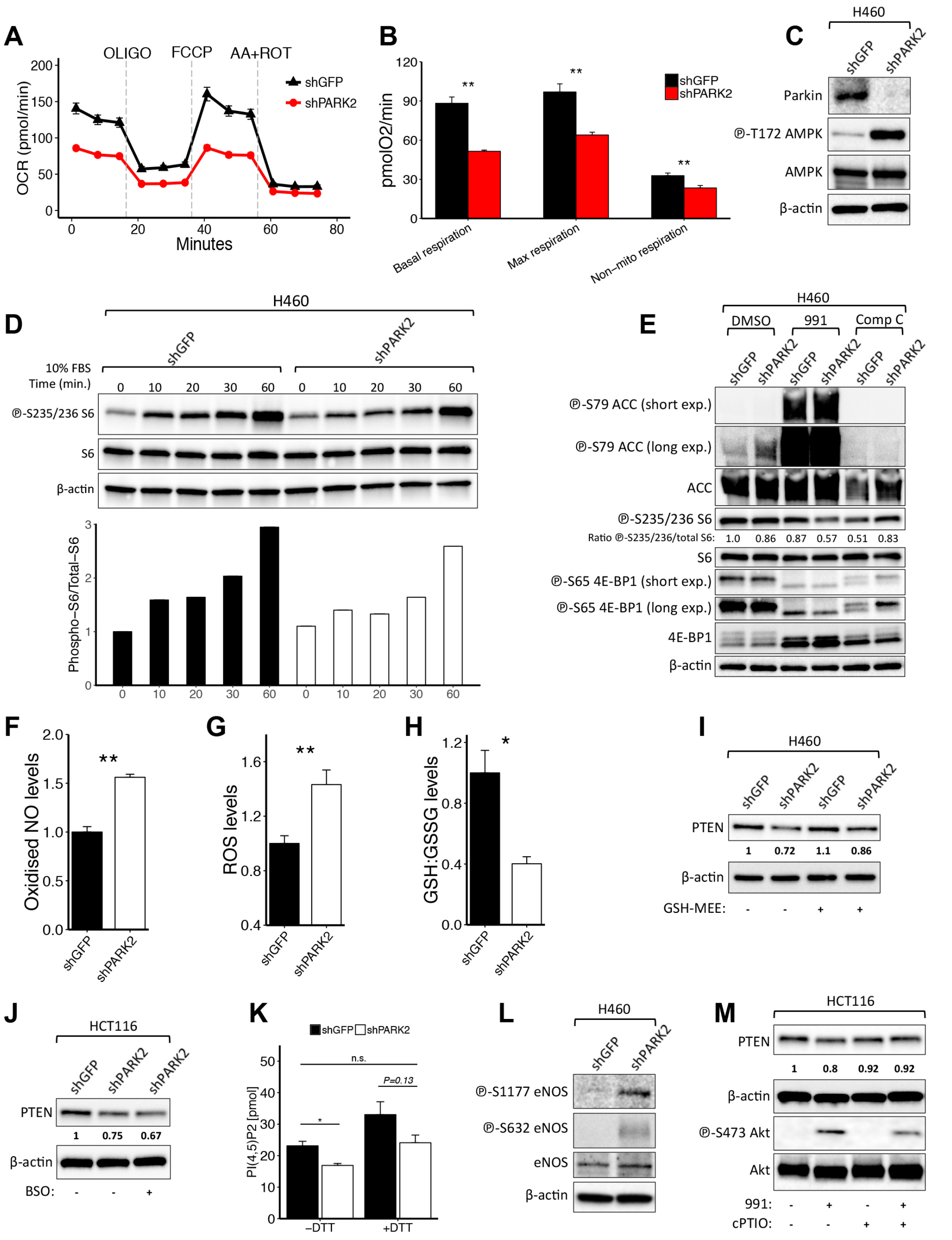

Figure S4, related to Figures 4 and 5. *PARK2* depletion enhances energy depletion-mediated oxidative and nitrosative stress.

(A) Seahorse analysis of oxygen consumption rate (OCR) in shGFP and shPARK2-expressing H460 cells, following sequential injection of oligomycin, FCCP and antimycin A/rotenone (n=6).

(B) Oxygen consumption rates of basal ($p = 2.1x10^{-5}$), maximal ($p = 5x10^{-4}$) and non-mitochondrial respiration (p = 0.006) of shGFP and shPARK2 H460 cells.

Immunoblotting analysis of

(C) Phospho-AMPK (T172), between shGFP and shPARK2 H460 cells.

(D) Phospho-S6 (S235/236) following 10% FBS stimulation (top), with densitometric analysis of immunoblotting (bottom), and

(E) AMPK and mTORC1 activation markers following treatment with DMSO, AMPK activator 991 (20 μM for 5 hours) and AMPK inhibitor Compound C (20 μM for 5 hours).

(F) Oxidized nitric oxide (NO) levels upon *PARK2* depletion in H460 cells ($p = 9x10^{-4}$).
(G) Reactive oxygen species (ROS) levels following 2 hours menadione treatment (20uM) ($p = 0.023$), and
(H) GSH:GSSG levels between shGFP and shPARK2 H460 cells ($p = 0.018$).
Immunoblotting analysis of
(I) shGFP and shPARK2 H460 cells with or without treatment with 5mM glutathione reduced ethyl ester (GSH-MEE) for 24 hours,
(J) shGFP and shPARK2 HCT116 cells with or without treatment with 500 μM BSO for 16 hours.
(K) PTEN activity of shGFP or shPARK2 HCT116 cells in the absence (-) or presence (+) of DTT (50 mM) [shGFP (-DTT) vs. shPARK2 (-DTT); $p = 0.02$, shGFP (+DTT) vs. shPARK2 (+DTT); $p = 0.13$, shGFP (-DTT) vs. shPARK2 (+DTT); $p = 0.75$].
Immunoblotting analysis of
(L) shGFP and shPARK2 H460 cells,
(M) HCT116 cells following treatment with the allosteric AMPK activator 991 (20 μM) for 5 hours with or without co-treatment with cPTIO (100 μM) for the same period. Data are represented as mean ±SEM (n.s.: not significant, $*p < 0.05$, $**p < 0.01$, 2-tailed *t* test).

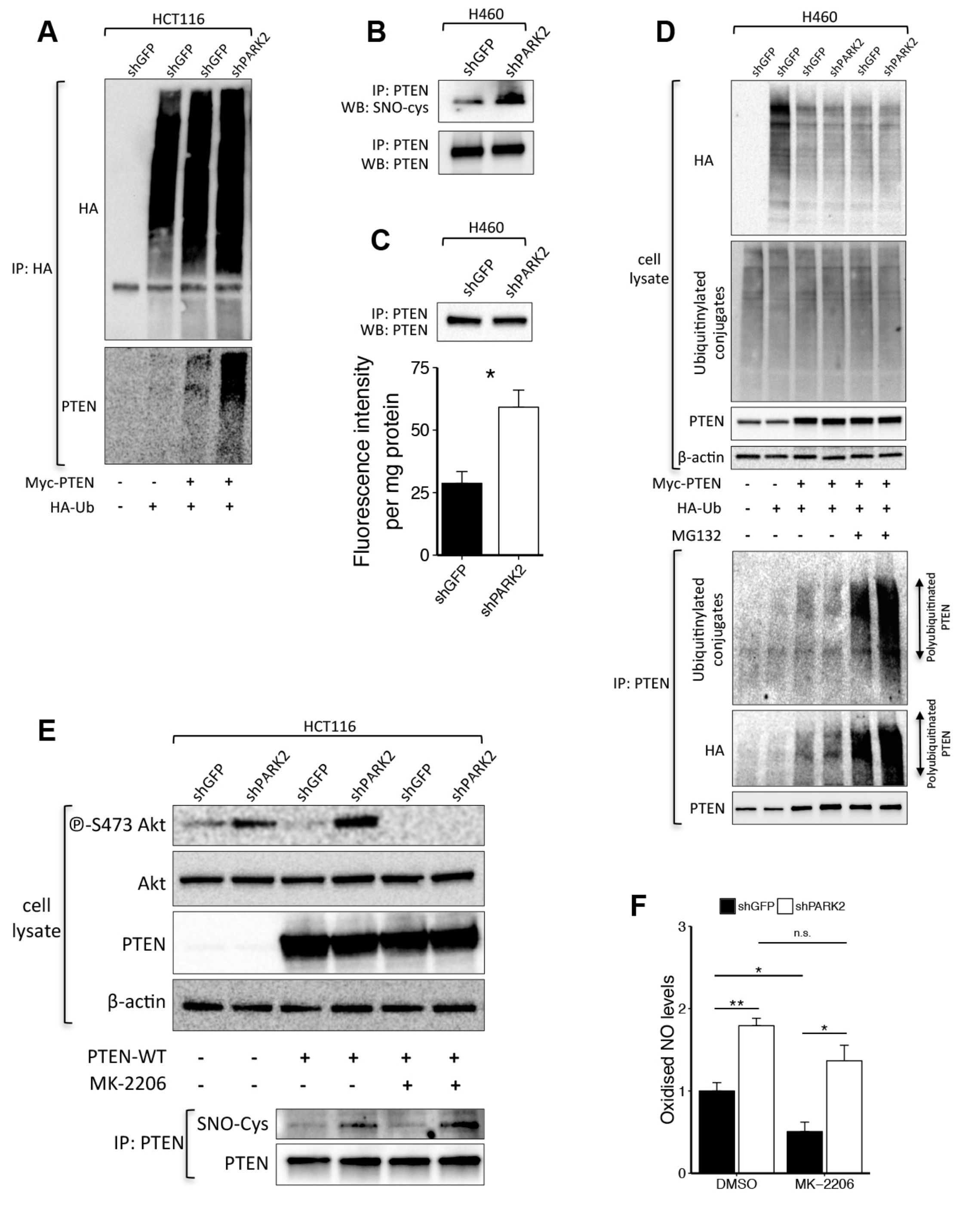

Figure S5, related to Figure 6. *PARK2* depletion promotes PTEN inactivation by S-nitrosylation and ubiquitination.

(A) Immunoblotting analysis of anti-HA immunoprecipitates (IP) derived from HA-ubiquitin (Ub) and Myc-tagged *PTEN* transfected shGFP and shPARK2 HCT116 cells.

(B) Immunoblotting analysis of Anti-PTEN immunoprecipitates (IP) derived from MYC-tagged transfected *PTEN* shGFP and shPARK2 H460 cells.

(C) Fluorometric measurement of S-nitrosylated PTEN between shGFP and shPARK2 H460 cells ($p = 0.021$).

(D) Immunoblotting analysis of whole-cell lysates and anti-PTEN immunoprecipitates (IP) derived from HA-ubiquitin (Ub) and Myc-tagged *PTEN* transfected shGFP and shPARK2 H460 cells. Where indicated cells were treated with MG132 (10 μM) for 6 hours before collection.

(E) Immunoblotting analysis and anti-PTEN immunoprecipitates derived from WT PTEN-expressing shGFP and shPARK2 HCT116 cells following 24 hour treatment with Akt inhibitor MK-2206 (100 nM).

(F) Oxidized nitric oxide (NO) levels of HCT116 cells following 24 hour treatment with DMSO or Akt inhibitor MK-2206 (100 nM) [shGFP (DMSO) vs. shPARK2 (DMSO); $p = 0.004$, shGFP (MK-2206) vs. shPARK2 (MK-2206); $p = 0.018$, shGFP (DMSO) vs. shGFP (MK-2206); $p = 0.033$, shPARK2 (DMSO) vs. shPARK2 (MK-2206); $p = 0.112$]. Data are represented as mean ±SEM (n.s.: not significant, *$p < 0.05$, **$p < 0.01$, 2-tailed *t* test).

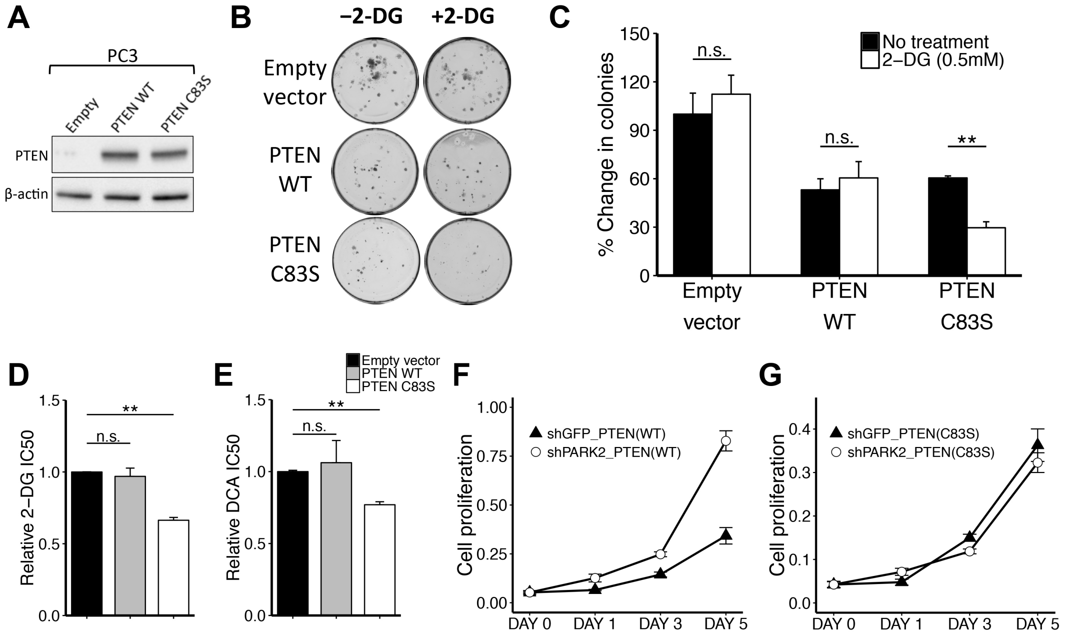

Figure S6, related to Figure 6. PTEN S-nitrosylation supports cell survival and proliferation under conditions of energy deprivation.

(A) Immunoblotting analysis of whole-cell lysates of PC3 cells expressing empty-vector, WT or S-nitrosylation-resistant (C83S) mutant *PTEN*.

(B-C) Clonogenic assays performed in 2-DG-treated (0.5 mM) or untreated PC3 cells expressing empty-vector (EV), wild-type (WT) or S-nitrosylation-resistant (C83S) mutant *PTEN* [EV (-DTT) vs. EV (+DTT); p = 0.52, *PTEN* WT (-DTT) vs. *PTEN* WT (+DTT); p = 0.577, *PTEN* C83S (-DTT) vs. *PTEN* C83S (+DTT); p = 0.001].

(D) 2-DG and (E) DCA IC_{50} values for PC3 cells expressing empty-vector, WT or C83S mutant *PTEN* (2-DG IC_{50}: EV vs. *PTEN* WT; p = 0.622, EV vs. *PTEN* C83S; p = 6.9×10^{-5}, DCA IC_{50}: EV vs. *PTEN* WT; p = 0.708, EV vs. *PTEN* C83S; p = 5.3×10^{-4}).

Cell proliferation assays of shGFP or shPARK2 PC3 cells co-transfected with (F) WT or (G) C83S mutant *PTEN* (PTEN WT: shGFP vs. shPARK2; p = 4.7×10^{-5}, PTEN C83S: shGFP vs. shPARK2; p = 0.512, 2-way Anova). Data are represented as mean ±SEM (n.s.: not significant, *p < 0.05, **p < 0.01, 2-tailed *t* test).

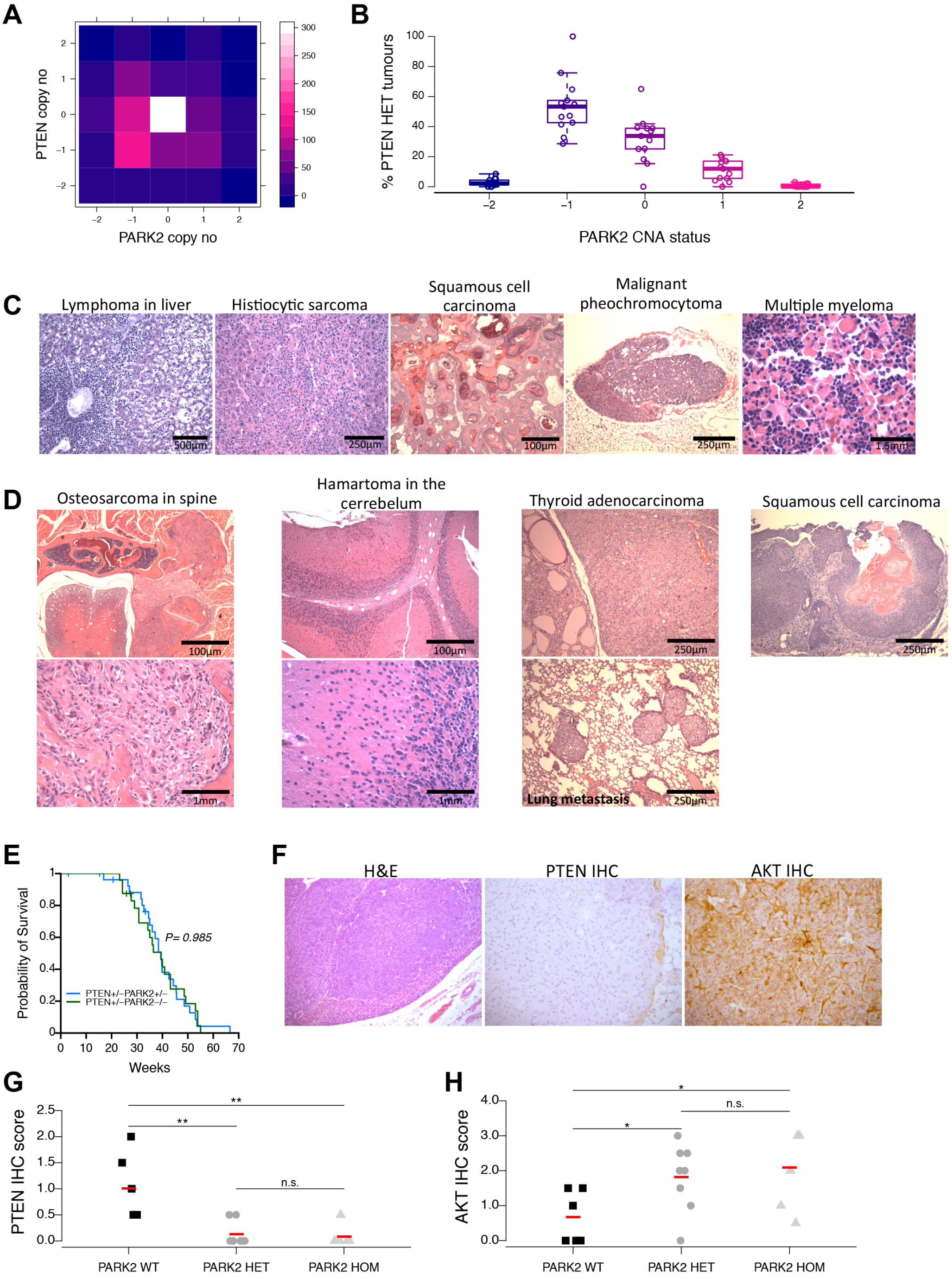

Figure S7, related to Figure 7. *PARK2* loss and *PTEN* loss display striking cooperativity to promote tumorigenesis in vivo.

(A) Image-plot indicating the co-existence of *PARK2* and *PTEN* copy number alterations (CNAs) across 995 cell lines from the Cancer Cell Line Encyclopedia (CCLE). The color panel indicates the number of cell lines for each combination and x- and y-axis indicate the CNA status of *PARK2* or *PTEN* genes, respectively.

(B) Box-plot indicating the fraction of *PARK2* CNAs of primary tumors or cancer cell lines with heterozygous deletion of *PTEN*. This analysis was performed on 1,953 specimens across 13 different cancer types from the TCGA database. Each point represents the percentage of a different tumor type with the respective copy number alteration in *PARK2*. A point was also added to represent the fraction of *PARK2* CNAs in cancer cell lines with heterozygous deletion of *PTEN* as reported in the CCLE database e.g. 17/312 (5.4%) have homozygous

deletion, 145/312 (46.5%) have heterozygous deletion, 79/312 (25.3%) have retention, 66/312 (21.2%) have 1-copy gain and 5/312 (1.6%) have 2-copy number gain of the *PARK2* gene.

(C) Representative H&E-stained sections of various tumor types found in *Pten*$^{+/-}$ mice with heterozygous and

(D) homozygous *Park2* deletion. Scale bar is 0.1-1.5 mm.

(E) Kaplan–Meier survival plot of *Pten*$^{+/-}$ mice bearing heterozygous (blue) (n=27) or homozygous (green) (n=24) *Park2* deletion, indicating that there is no statistical difference in survival between the two mouse cohorts (p = 0.985, Log-rank test).

(F) Representative H&E (x100), PTEN (x200) and AKT (x200) stained sections of an adrenal phaechromocytoma found in a *Pten*$^{+/-}$ *Park2*$^{+/-}$ mouse.

(G) Quantification of IHC scores of PTEN and (H) AKT protein expression in mouse tissue sections from tumor lesions found in *Pten*$^{+/-}$ mice with WT, HET or HOM deletion of *Park2* (PTEN IHC: *Park2* WT vs. HET p = 0.003, *Park2* WT vs. HOM p = 0.007, AKT IHC: *Park2* WT vs. HET p = 0.033, *Park2* WT vs. HOM p = 0.027, 2-tailed *t*-test).

SUPPLEMENTAL TABLES

Table S1. Related to Figure 1. Number and type of tumors used to report the frequency of *PARK2* DNA copy number loss from the TCGA dataset. Table S1 is provided as a Microsoft Excel file.

Table S2. Related to Figure 1. Number and type of tumors that showed significantly lower *PARK2* mRNA expression compared to their normal counterparts. Table S2 is provided as a Microsoft Excel file.
